# LLM-Guided Adaptive Cost Calibration in Active Learning for Parkinson’s Disease Biosignals

**DOI:** 10.3390/ijms27219542

**Published:** 2026-10-25

**Authors:** Burcu Gülez, Ali Güneş, Ersoy Kocabıçak

**Affiliations:** 1 Faculty of Medicine, Istanbul Atlas University, Istanbul 34408, Türkiye; ersoy.kocabicak@atlas.edu.tr; 2 Department of Software Engineering, Faculty of Engineering and Natural Sciences, Istanbul Atlas University, Istanbul 34408, Türkiye

**Keywords:** Parkinson’s disease, artificial intelligence, active learning, cost-sensitive learning, large language model, digital biomarkers, *α*-synuclein, gait analysis, speech biomarkers, trustworthy AI

## Abstract

Confirmatory labels for Parkinson’s disease (PD), from expert examination or, prospectively, biological assays, are costly, and missed cases and false alarms carry unequal consequences. ClinRisk-LLM is a framework for label-efficient, cost-aware learning from gait and speech signals in which a large language model (LLM) translates a predefined safety specification into a frozen policy setting the positive-class weight of a support vector machine and the priority of potential missed cases for annotation. Cost-sensitive training, risk-aware querying and the LLM policy were ablated on gait (n=165) and speech (n=252) data and benchmarked against post hoc thresholding, a refer-all policy, held-out gait studies and an independent speech cohort. With half of the labels, cost-sensitive active learning reduced the 4:1 asymmetric cost by 34.1% (gait) and 5.7% (speech) relative to standard active learning, at the expense of specificity. ClinRisk-LLM translated the predefined safety specification into a frozen, auditable policy that adjusted risk settings during label acquisition. Component-wise analyses indicated that classifier weighting accounted for most of the observed cost reduction, while the incremental benefit of LLM-guided adaptation varied across datasets and comparison settings. A threshold-tuned symmetric model came within 1.3 cost points for gait, and gait remained roughly level with referring everyone. ClinRisk-LLM reached a gait cost of 47.60 versus 46.10 for fixed cost-sensitive learning; 40 policies generated across three LLMs from a reconstructed specification yielded gait costs of 52.1–75.8. Applications to molecular triage remain prospective. Code and data availability details are provided in the Data Availability Statement.

## 1. Introduction

Parkinson’s disease (PD) is the second most common neurodegenerative disorder and among the fastest-growing neurological causes of disability worldwide [1,2]. Models based on the Global Burden of Disease 2021 data place the number of people living with PD at about 25.2 million by 2050 [3,4]. This projection is well above earlier estimates and adds urgency to the search for scalable ways to assess the disease. PD is marked by the progressive loss of dopaminergic neurons in the substantia nigra pars compacta and by misfolded, aggregated α-synuclein in Lewy pathology. Its pathogenesis cannot be reduced to either process alone. Mitochondrial, lysosomal and endosomal dysfunction, oxidative stress, and altered immune responses are also involved [5,6].

Loss of nigrostriatal dopaminergic signaling contributes to bradykinesia, rigidity, resting tremor and gait impairment. Pathology outside this system helps to account for the varied non-motor manifestations [5]. Available treatments are primarily symptomatic. Levodopa-based therapy remains central, with device-aided interventions reserved for selected patients with more advanced disease [7]. Although recent research frameworks incorporate α-synuclein pathology, neurodegeneration and genetic factors, diagnosis in routine practice is still largely clinical [8]. Substantial nigrostriatal degeneration may already be present when the characteristic motor syndrome becomes apparent [9]. Objective measures may therefore complement periodic clinical examination. Such measures do not establish diagnostic or clinical utility on their own.

The field is now moving toward a biological definition of PD. Research frameworks such as the SynNeurGe criteria and the neuronal α-synuclein disease integrated staging system anchor the disease in α-synuclein pathology, dopaminergic neurodegeneration and genetic risk [10,11]. Cerebrospinal fluid α-synuclein seed amplification assays (SAAs) detect misfolded α-synuclein in most people with sporadic PD, but sensitivity varies with genotype and clinical phenotype [12]. Pathogenic variants in genes such as *LRRK2* and *GBA1* and many common risk loci further shape molecular heterogeneity [13]. These confirmatory measures are informative but costly, invasive or limited in availability. Low-cost motor and speech signals are downstream readouts of the same pathology. In future, they might help decide which individuals should be referred for expensive expert or molecular confirmation. This is a prospective application that the present study does not test. Such a triage role would require a learning method that uses few confirmed labels and explicitly prices the consequences of a missed case.

Wearable sensors, portable devices and smartphones make it possible to measure disease-related physiological and behavioral changes repeatedly and without invasive procedures [14]. Gait and speech are useful examples, although they differ substantially as signals. Gait changes in PD affect pace, rhythm, variability, asymmetry and dynamic stability. Common findings include reduced speed and stride length, increased stride-to-stride variability, and altered arm or trunk motion [15]. Hypokinetic dysarthria affects several parts of speech production, including phonation, articulation, prosody, respiration and resonance. These changes can be described through intensity, fundamental frequency, jitter, shimmer, articulation measures and speech timing [9].

Computational PD research now draws on acoustic signals, movement measurements, neuroimaging and biological markers [16]. Most studies of digitally recorded motor data still distinguish people with PD from healthy controls. Support vector machines and related conventional classifiers are often used [17]. Strong accuracy in a retrospective case–control dataset, however, says little about performance in population screening. A sound evaluation requires participant-level separation and test-independent preprocessing. It also requires explicit reporting of sensitivity and specificity. Label acquisition is another practical constraint. Moreover, false negatives (FNs) and false positives (FPs) do not necessarily have comparable consequences.

Pool-based active learning (AL) addresses labeling effort by choosing which observations should be annotated [18]. Cost-sensitive classification addresses a different problem. It assigns different penalties to different errors [19]. The two mechanisms must be evaluated separately. Acquisition order no longer affects the fitted model once the entire pool has been labeled. Any full-budget difference therefore comes from classifier weighting or threshold choice rather than from AL.

### 1.1. AI-Enabled Computational Assessment from Parkinson’s Gait and Speech

Quantitative gait analysis represents PD-related changes through pace, rhythm, variability, bilateral asymmetry and dynamic stability [15]. The PhysioNet database contains vertical ground-reaction-force recordings. It has supported classical event-derived analyses and supervised learning studies [20,21]. Speech-based PD classification spans perturbation measures, cepstral coefficients, wavelet representations and feature-selection pipelines. These measures reflect impairment across several components of speech production [9]. The Sakar dataset was designed for this high-dimensional acoustic setting. Its repeated recordings require participant-level aggregation or grouping to prevent recording-level leakage [22,23].

Results from retrospective public datasets are easily overstated. High case–control accuracy is not evidence of screening performance, particularly if recordings from the same participant cross data partitions. We therefore regard participant-level splitting, test-independent preprocessing and specificity reporting as necessary whenever sensitivity is given priority.

### 1.2. Active Learning and Asymmetric Error Cost

Pool-based AL iteratively trains a model on a labeled set, ranks an unlabeled pool and requests labels for selected observations [18]. Margin sampling is natural for SVMs, while batch diversity can reduce redundant queries. Cost-sensitive learning instead changes the training objective or decision threshold so errors incur unequal loss [19]. These mechanisms are distinct: an acquisition rule controls which labels are obtained, whereas class weights control how obtained labels shape the classifier.

This distinction shapes our experimental design. Comparing symmetric AL directly with a class-weighted AL model would mix the effect of acquisition with the effect of training. We therefore include query-only and cost-only arms. We also avoid interpreting full-budget differences as AL gains, because all acquisition histories lead to the same labeled pool at that point.

### 1.3. LLMs as Adaptive Policy Generators

LLM-guided risk calibration has also been studied in cost-sensitive active learning for UAV weed detection. AdaRisk-Agent adjusted the risk coefficient from evolving scene statistics and reduced FNR across distinct multispectral scenes [24]. This work provides a cross-domain precedent for using an LLM as a structured calibration component. The LLM in ClinRisk-LLM has a deliberately narrow role. It receives a restricted, test-independent specification and returns an ordered calibration policy. That policy is stored before evaluation and replayed without further LLM calls. It never labels an observation or predicts a participant’s diagnosis. We compare it with an independently written adaptive rule to assess its value beyond ordinary threshold logic.

### 1.4. Aims and Contributions

Here, we study uncertainty–diversity querying in gait and speech data. The central question is whether risk-sensitive querying adds anything beyond class-weighted training when only part of the pool is labeled. We also measure the associated sensitivity–specificity trade-off and compare the AL models with cost-matched classifiers trained on the full pool. The study is methodological and does not assess clinical diagnostic utility.

Our contribution is a controlled, cross-modality ablation rather than a new biomarker or diagnostic device. The design uses label-blind initialization and prioritizes negative-side observations that may be false negatives. Every method is evaluated with the same cost. Query selection and classifier weighting are tested separately and together. Cost-matched full-pool models serve as references. We also introduce ClinRisk-LLM. It uses an LLM once, before evaluation, to translate a restricted safety specification into a fixed adaptive policy. It does not use the LLM to annotate data or classify individual participants.

The specific objectives are the following:

1. To quantify cost-sensitive AL relative to standard AL under one common, prespecified evaluation loss.
2. To separate the contributions of risk-sensitive query selection and class-weighted classifier training.
3. To characterize learning behavior across the complete 10–100% annotation-budget trajectory in two structurally different biosignal modalities.
4. To compare partial-label models with full-pool symmetric, class-weighted and cost-threshold references without attributing full-budget differences to query order.
5. To quantify the downstream confirmatory-testing burden of the sensitivity-weighted operating points against a trivial refer-all policy, and to explain the difference between modalities.
6. To assess robustness across independently recorded cohorts and across repeated generations of the LLM-derived policy.

## 2. Results

This section presents the experimental findings in three stages. The first analysis traces changes in cost, FNR and specificity across the annotation budget. The second separates the contributions of classifier weighting and risk-aware querying. The final analysis compares the proposed policies with full-pool references, fixed cAL and the deterministic Adaptive Rule. Together, these comparisons characterize both the source and the consistency of the observed performance differences.

### 2.1. Effect of the Annotation Budget

We first evaluated all component-wise arms at 50% labeling, the primary restricted-budget operating point. This comparison measures whether the proposed mechanisms improve the common asymmetric objective before the complete development pool is annotated.

**Figure 1:**
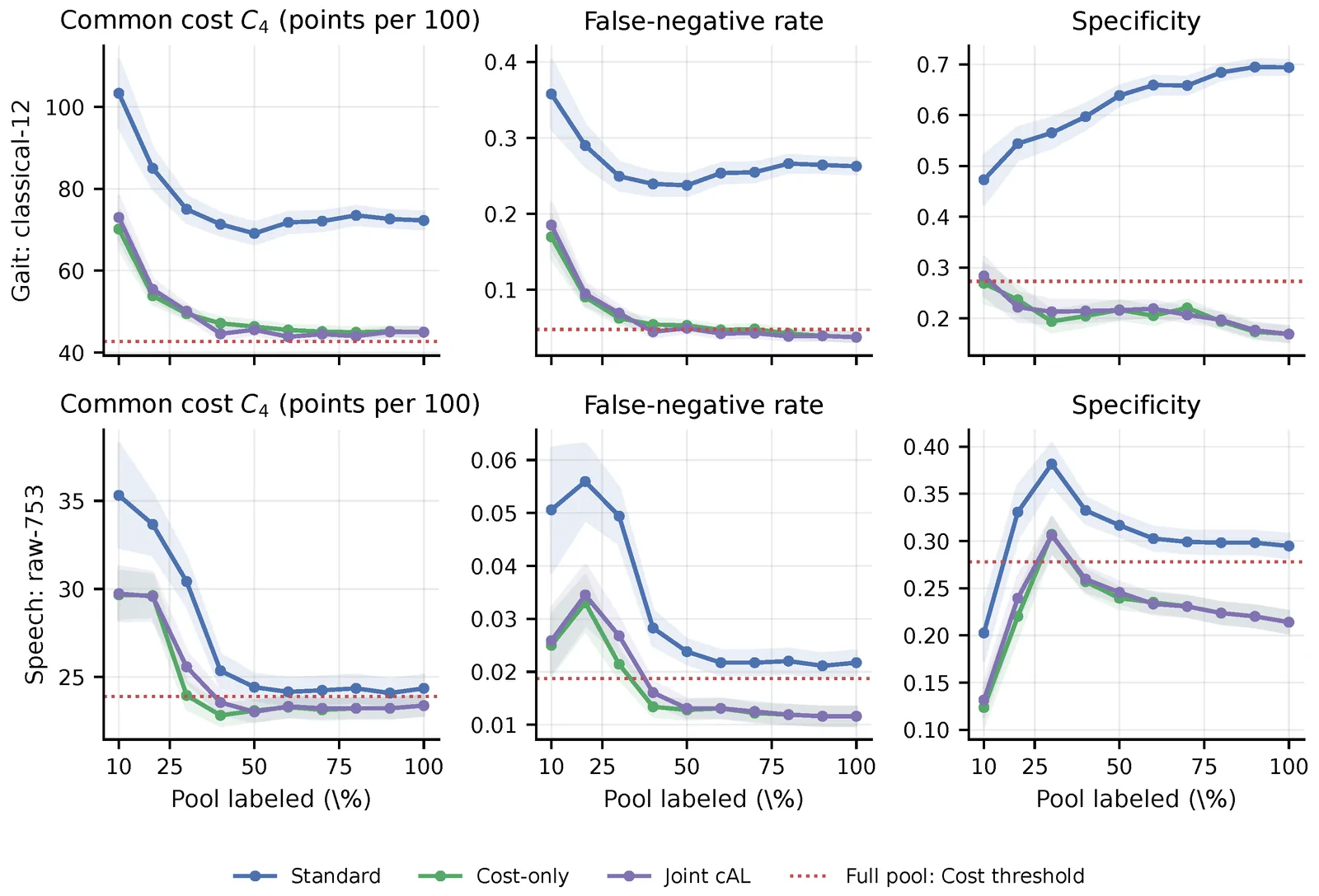
Budget-dependent $C_{4}$, FNR and specificity for gait and speech. Colors identify Standard AL, cost-only training, and joint cAL as shown in the legend; the red dotted line marks the full-pool cost-threshold reference. Shading denotes ±1 standard error.

The largest difference in Table 1 occurs for gait. Joint cAL lowers $C_{4}$ from 69.10 to 45.57 relative to Standard AL. This corresponds to a reduction of 34.1%. FNR also falls from 0.237 to 0.049. Specificity decreases from 0.639 to 0.216. The cost reduction therefore comes at the price of more false positives. The effect is smaller for speech. Here, $C_{4}$ falls from 24.42 to 23.02, a reduction of 5.7%. FNR falls from 0.024 to 0.013. Cost-only training gives a nearly identical speech cost of 23.09. This suggests that weighting explains almost all of the change. The preferred method therefore depends on the target metric. Symmetric and query-only models retain an advantage in specificity, precision or balanced accuracy. Random acquisition was the weakest acquisition rule for speech (27.27 vs. 24.42 for standard querying) and worse than standard querying for gait (77.07 vs. 69.10). Post hoc thresholding of a symmetric model, with the threshold tuned only on the labeled half, came within 1.3 points of joint cAL for gait (46.83 vs. 45.57). For speech, it came closer than symmetric training but remained above joint cAL (24.13 vs. 23.02). Across the gait trajectory, the threshold-tuned model was within 4.2 cost points of joint cAL at every budget. It was better at 10% (70.4 vs. 73.0) and worse at 40% (48.8 vs. 44.6). Most of the benefit of cost-sensitive training can therefore be obtained by moving the threshold of a symmetric model. The refer-all reference qualifies these reductions. Labeling every participant PD gives $C_{4}=44.00$ for gait and 25.33 for speech. Under the 4:1 loss, Standard AL is therefore worse than referring everyone for gait (69.10), and joint cAL is 1.57 points above the reference. For speech, joint cAL is 2.31 points below the reference. Section 2.4 and Section 2.5 examine what this means for downstream testing and why the modalities differ. Figure 1 places this 50% comparison in context by following the three metrics over the full annotation trajectory.

Most of the cost reduction occurs well before the full pool is labeled (Figure 1). In gait, the cost-sensitive curves separate from Standard AL early and level off at roughly 40–50% labeling. From 40% labeling onward, joint cAL usually remains just below cost-only training. The two curves are almost indistinguishable for speech, leaving little evidence that risk-sensitive querying adds to class weighting in this modality. The dotted line is the cross-validated full-pool cost-threshold reference, and the query-only result omitted from the plot is reported in Table 1. Cost alone cannot reveal which type of error has changed, so we next examine sensitivity and specificity directly.

### 2.2. Analysis of the Effects of Classifier Weighting and Query Strategy

**Figure 2:**
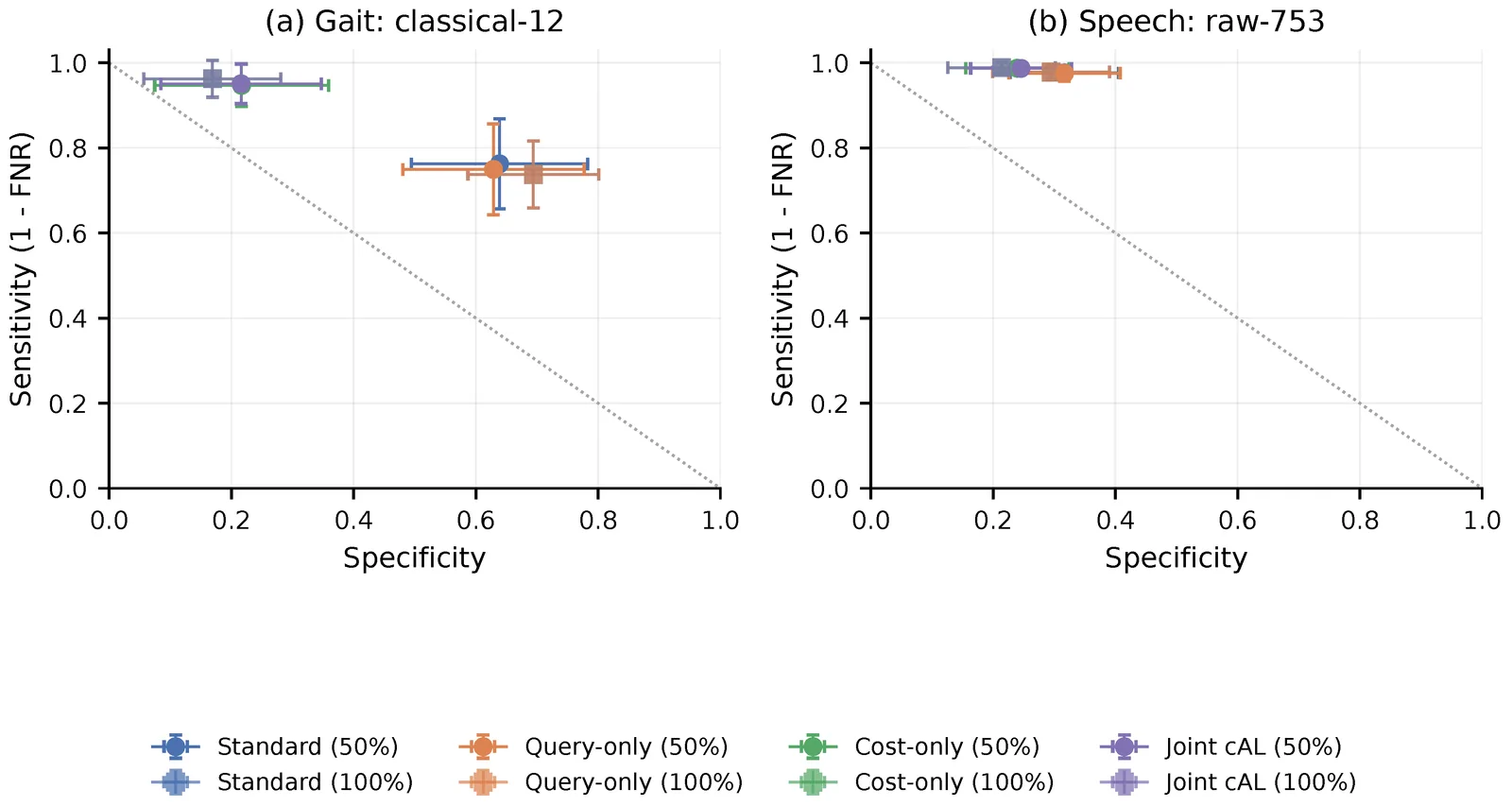
Sensitivity–specificity operating points at 50% and full labeling, with error bars showing one split-level standard deviation. The dashed gray diagonal is a visual guide; colors and marker shapes identify methods and budgets as shown in the legend.

To determine whether lower $C_{4}$ reflects improved discrimination or a change in operating point, we compared sensitivity and specificity at 50% and full labeling. Figure 2 first locates each method in this two-dimensional operating space. The component arms and their mean confusion matrices are then used to identify the source of the observed shifts.

The operating points in Figure 2 make the trade-off clear. Class-weighted gait models move toward high sensitivity and low specificity, while the query-only and standard symmetric models stay close to one another. The speech models cluster in a region of high sensitivity and modest specificity. Their small cost differences should therefore not be read as large gains in discrimination. The diagonal is included only as a visual guide.

Querying by itself did not improve on symmetric AL. At 50% labeling, the query-only cost was 72.47 rather than 69.10 for gait. It was 24.69 rather than 24.42 for speech. Weighting produced the main reduction. After weighting, risk-sensitive querying lowered mean gait cost from 46.33 to 45.57, a further reduction of 1.6%. This gain was not consistent across splits. Joint cAL had a lower cost than cost-only training in 40% of the gait splits, tied in 13% and was worse in 47%, with a median paired difference of zero (Table 2). Speech cost changed by only 0.3%, from 23.09 to 23.02, and the two arms were tied in 87% of splits. The additional query effect is therefore not reliable for gait and negligible for speech. By contrast, class weighting lowered gait cost in 87% of splits.

**Figure 3:**
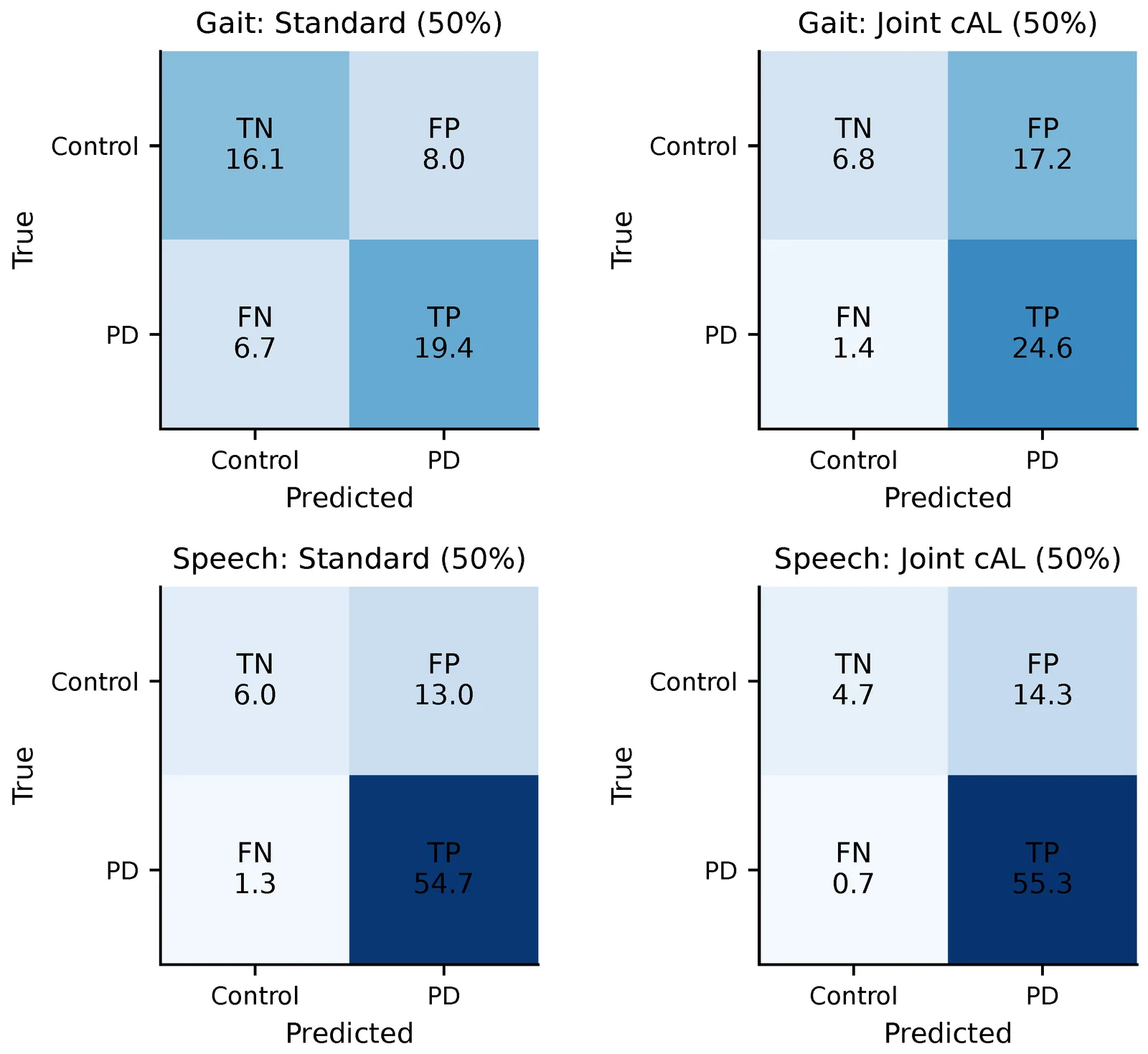
Mean 50%-budget confusion matrices for Standard AL and joint cAL across 60 splits. Cell color intensity represents the mean count, with darker blue indicating a higher count.

The mean confusion matrices in Figure 3 show where these changes in cost originate.

For gait, joint cAL turns many false negatives into true positives, but it also turns a sizeable number of true negatives into false positives (Figure 3). The change is less pronounced for speech because Standard AL already yields few false negatives in this case-enriched cohort. These matrices support a narrower interpretation of the improvement: the classifier is better aligned with the chosen cost, but it is not uniformly more accurate. The next comparison asks whether this pattern remains when all pool labels are available and when the risk weight is allowed to adapt.

### 2.3. Comparison with Reference and Adaptive Policies

We next compared the proposed cost-sensitive models with symmetric, class-weighted and cross-validated cost-threshold classifiers trained on the complete pool. This experiment establishes the terminal reference performance and prevents full-budget differences from being attributed to query order. Table 3 reports the terminal metrics under the same evaluation loss.

**Figure 4:**
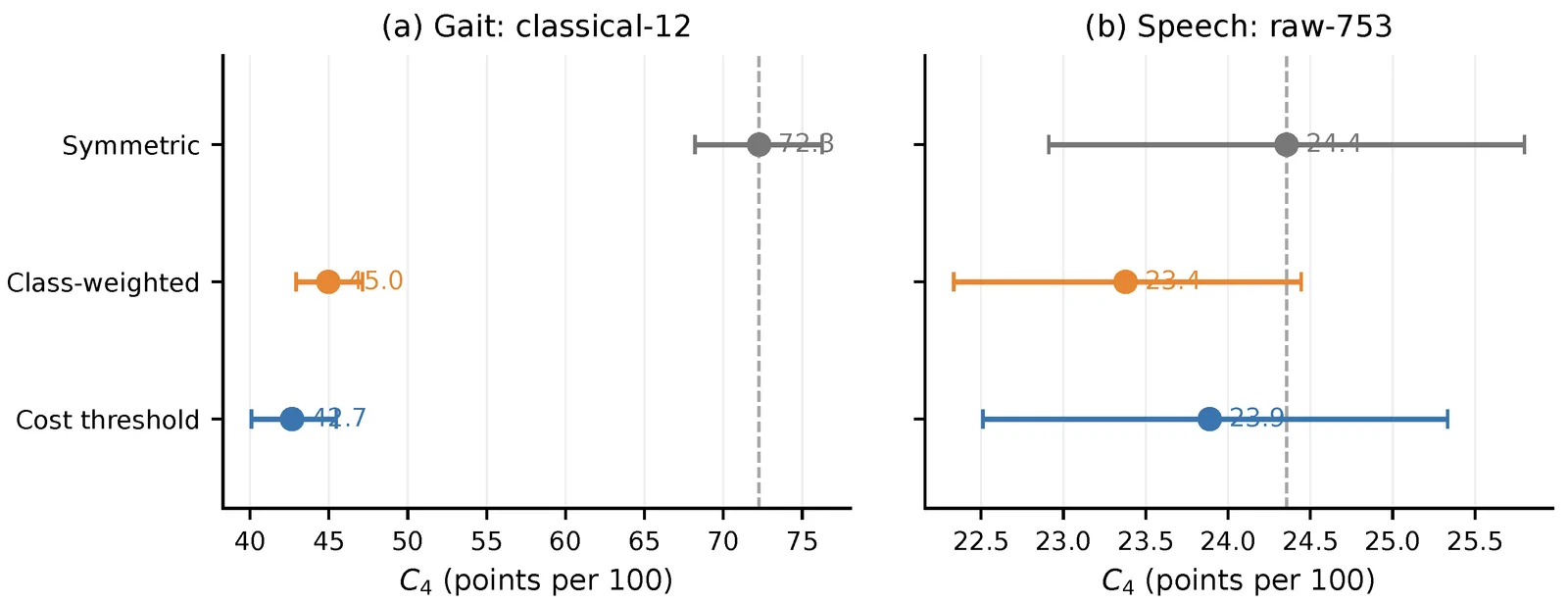
Full-pool $C_{4}$ means and descriptive bootstrap intervals for the three reference classifiers. Gray denotes the symmetric classifier, orange the class-weighted classifier, and blue the cost-threshold classifier; the vertical dashed line marks the symmetric mean.

Once the whole pool is labeled, query history no longer provides an advantage (Table 3). In gait, class weighting gives the lowest FNR, whereas the cost-matched threshold gives the lowest $C_{4}$ and recovers some specificity. The symmetric model still has the best balanced accuracy. For speech, class weighting gives the lowest FNR and cost, although the absolute difference from the symmetric and threshold references is small. Figure 4 presents the same costs with their descriptive bootstrap intervals, making both the ordering and the split-to-split uncertainty easier to compare.

The modality contrast is pronounced in Figure 4. Both cost-aware gait references lie well below the symmetric mean, while the three speech means occupy a narrow range. The cost-threshold model ranks first for gait and the class-weighted model for speech. Horizontal intervals describe variation across splits. The dashed line marks the symmetric mean.

The full-pool comparison defines the attainable terminal reference but does not test whether the risk weight should change during acquisition. We therefore compared Standard AL, fixed cAL, the independently specified Adaptive Rule and ClinRisk-LLM at the primary 50% labeling budget. Table 4 reports their outcomes under the same evaluation loss.

**Figure 5:**
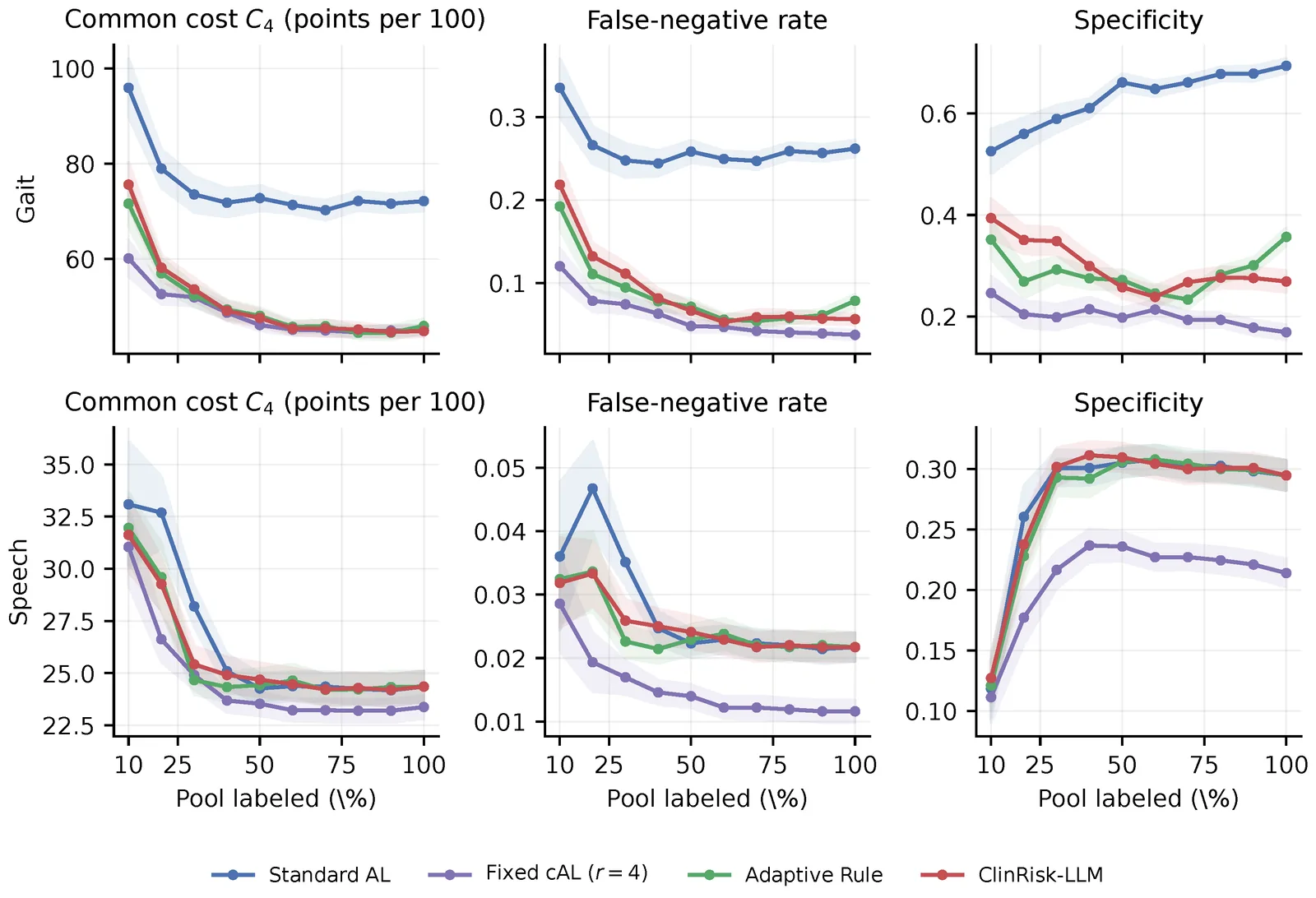
Learning curves for Standard AL, fixed cAL, Adaptive Rule and ClinRisk-LLM. Colored lines identify the policies in the legend; shaded bands show ±1 standard error.

Fixed cAL has the lowest gait cost and FNR in Table 4. Its values are 46.10 and 0.048, compared with 72.77 and 0.258 for Standard AL. ClinRisk-LLM reaches a gait cost of 47.60, between fixed cAL and Adaptive Rule (48.03). It preserves more specificity than fixed cAL, with values of 0.258 and 0.198. However, it does not beat fixed cAL on $C_{4}$, and its advantage over Adaptive Rule was not consistent across splits (lower cost in 52% of paired splits; Table 2). The ordering changes for speech. Fixed cAL has the lowest cost of 23.53 and the lowest FNR of 0.014. ClinRisk-LLM has the highest specificity of 0.310 and balanced accuracy of 0.643. Its mean risk weight is nearly symmetric at 1.03. We do not rank mean $r_{t}$, because it describes the policy’s response rather than predictive performance. Figure 5 shows when these policy differences emerge during acquisition.

**Figure 6:**
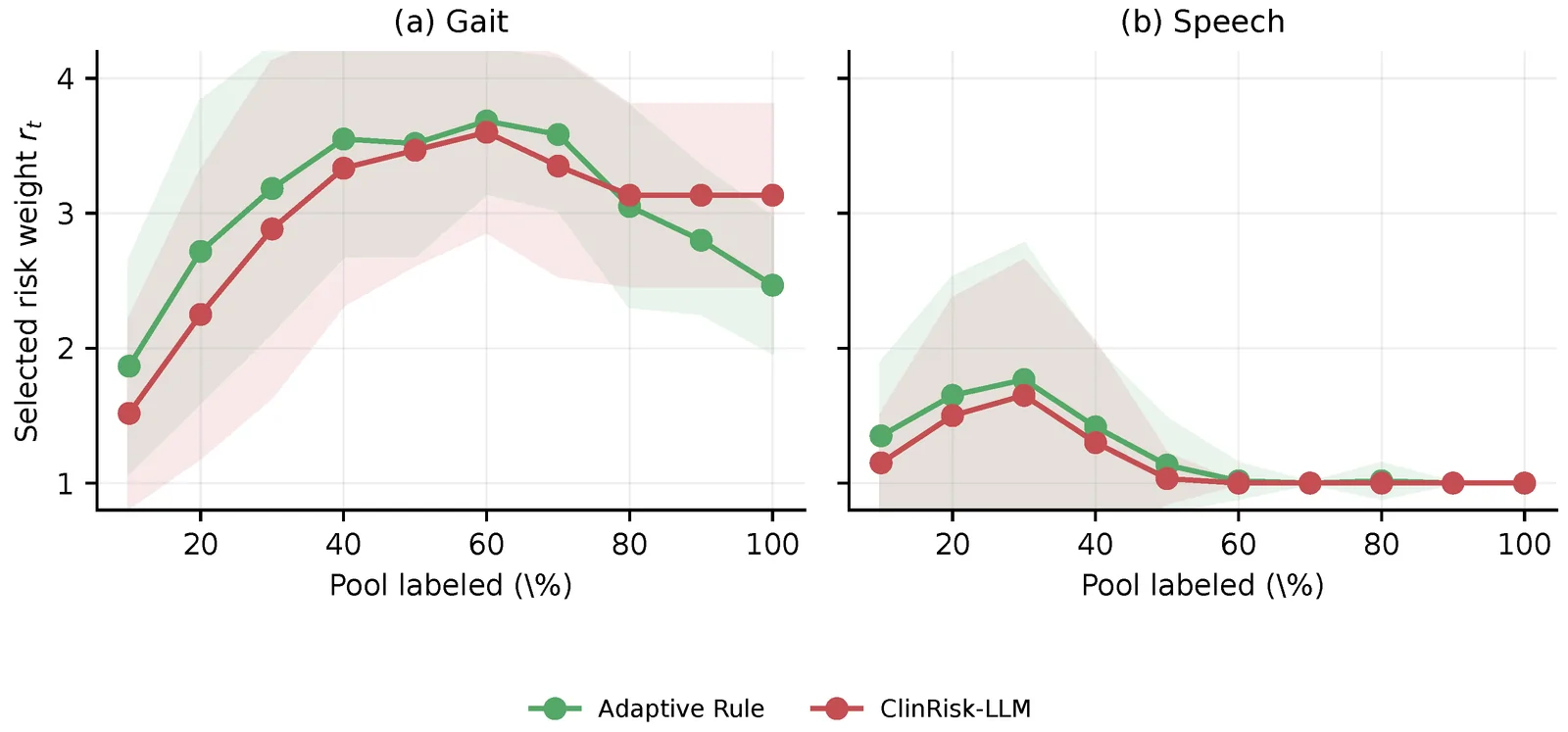
Mean adaptive risk-weight trajectories for the Adaptive Rule and ClinRisk-LLM, identified by the colored lines in the legend; shading shows one split-level standard deviation.

Fixed and adaptive cost sensitivity remain below Standard AL across much of the gait trajectory, and the three cost-sensitive policies follow similar paths (Figure 5). The speech curves stay close and their uncertainty bands overlap widely. ClinRisk-LLM responds differently to the two modalities, but there is no consistent optimization advantage. The risk-weight paths in Figure 6 help explain this difference.

Both adaptive policies maintain relatively high gait weights because the OOF signals continue to permit an emphasis on sensitivity (Figure 6). For speech, the specificity guard repeatedly pulls the weight back toward one, so training remains close to symmetric. The broad dispersion in parts of the trajectories also shows that the selected weights depend on the particular pool/test split.

The overall ranking depends on the metric. Cost-sensitive operating points perform best on $C_{4}$ and FNR, whereas symmetric models have the highest balanced accuracy in both modalities. At full labeling, gait balanced accuracy is 0.716 for the symmetric model, 0.566 with class weighting and 0.613 for the cost-threshold reference. The corresponding speech values are 0.637, 0.601 and 0.630. Under full-pool symmetric scoring, ROC-AUC remains about 0.79 for gait and 0.82 for speech. Taken together, these results point to a change in the decision operating point rather than stronger underlying class separation.

### 2.4. Downstream Testing Burden

Table 5 expresses the gait operating points as consequences for 1000 people who could be referred for confirmatory testing. In a specialist-referral setting with 30% prevalence, joint cAL refers 834 people and misses 15 PD cases. Standard AL refers 482 and misses 71. Joint cAL therefore detects about 56 additional cases at the cost of about 352 additional confirmatory tests, including 296 additional unnecessary tests. This corresponds to 6.2 additional total tests, or 5.2 additional unnecessary tests, per additional case found. Compared with referring all 1000, it avoids only 151 unnecessary tests, and its net benefit at $p_{t}=0.2$ is slightly lower than that of Standard AL (0.148 vs. 0.166). At population-screening prevalences, the burden is much larger. At 1% prevalence, joint cAL refers 786 people to find 9.5 cases (PPV 1.2%), and each additional case found relative to Standard AL costs about 223 extra total tests. At these low prevalences, net benefit at $p_{t}=0.2$ is negative for every operating point. No model would then be preferable to referring nobody under the 4:1 exchange rate.

For the fixed gait operating points, the FN:FP exchange rate at which joint cAL and Standard AL have equal mean transported cost is ΔFP/ΔTP. It is approximately 5.24:1 at 30% prevalence, 20.23:1 at 10% and 222.49:1 at 1%. Joint cAL is favored over Standard AL only above these ratios, under the constant-sensitivity/specificity assumption. This pairwise calculation does not establish superiority over refer-all, refer-none or other classifiers. It is a post hoc assessment of fixed predictions, not a new optimization of the policies for each utility.

**Figure 7:**
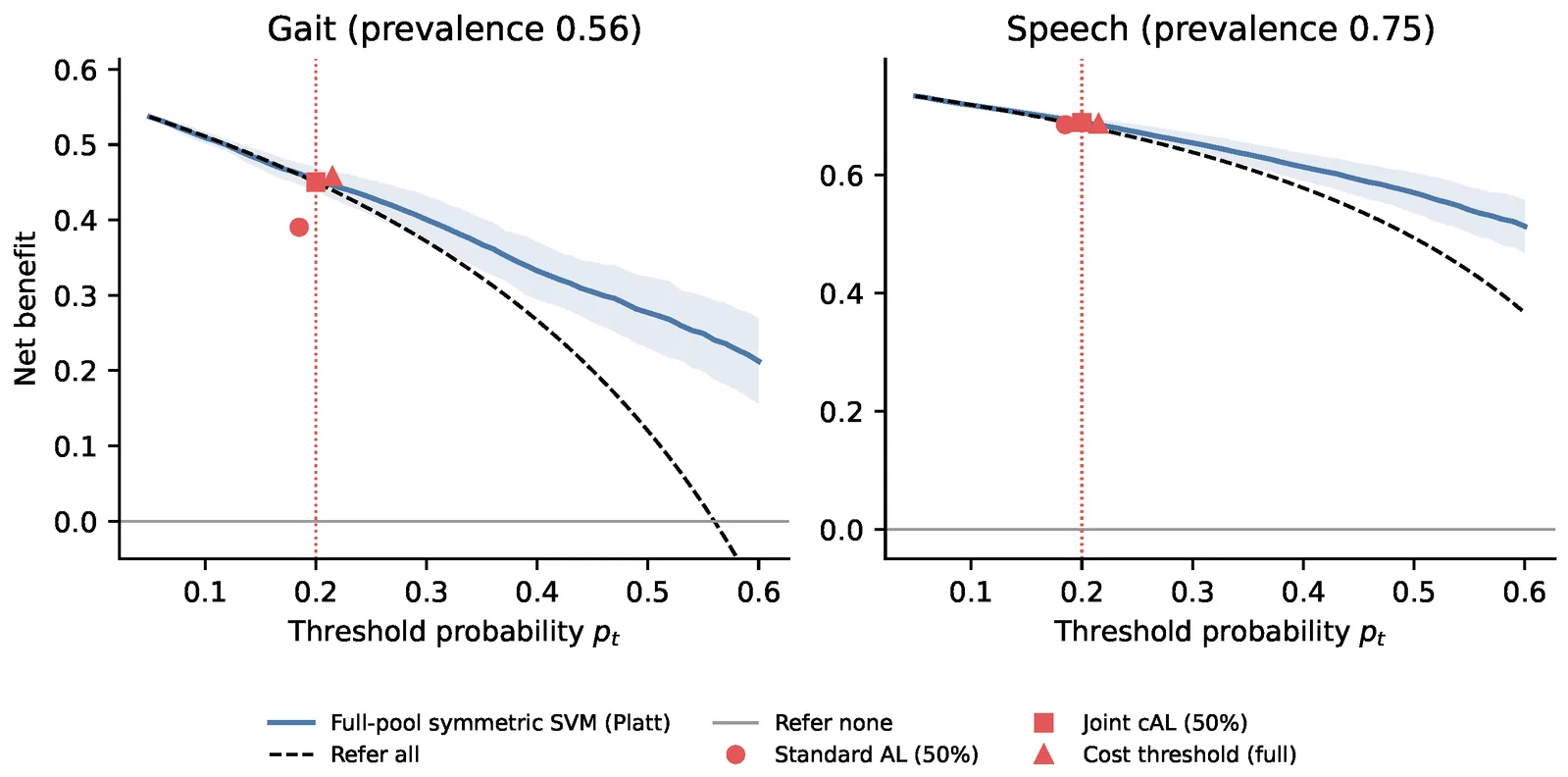
Decision curves for full-pool symmetric SVMs with Platt-scaled probabilities (mean ±1 split-level SD) against refer-all and refer-none. The blue curve and band show the SVM mean and split-level standard deviation; the black dashed curve is refer-all and the gray horizontal line is refer-none. Red markers show the indicated operating points at $p_{t}=0.2$ (circle: Standard AL; square: joint cAL; triangle: cost threshold), offset horizontally for legibility; the red dotted vertical line marks $p_{t}=0.2$.

The decision curves in Figure 7 lead to the same conclusion. At $p_{t}=0.2$, the full-pool symmetric SVM is almost indistinguishable from refer-all in both modalities (net benefit 0.454 vs. 0.450 for gait and 0.689 vs. 0.683 for speech). The biosignals add clear value only at higher thresholds, that is, when unnecessary confirmatory tests are weighted more heavily. At $p_{t}=0.4$, which corresponds to an FN:FP ratio of 1.5:1, the gait model has a net benefit of 0.332 compared with 0.267 for refer-all.

### 2.5. Why the Gain Differs Between Modalities

**Figure 8:**
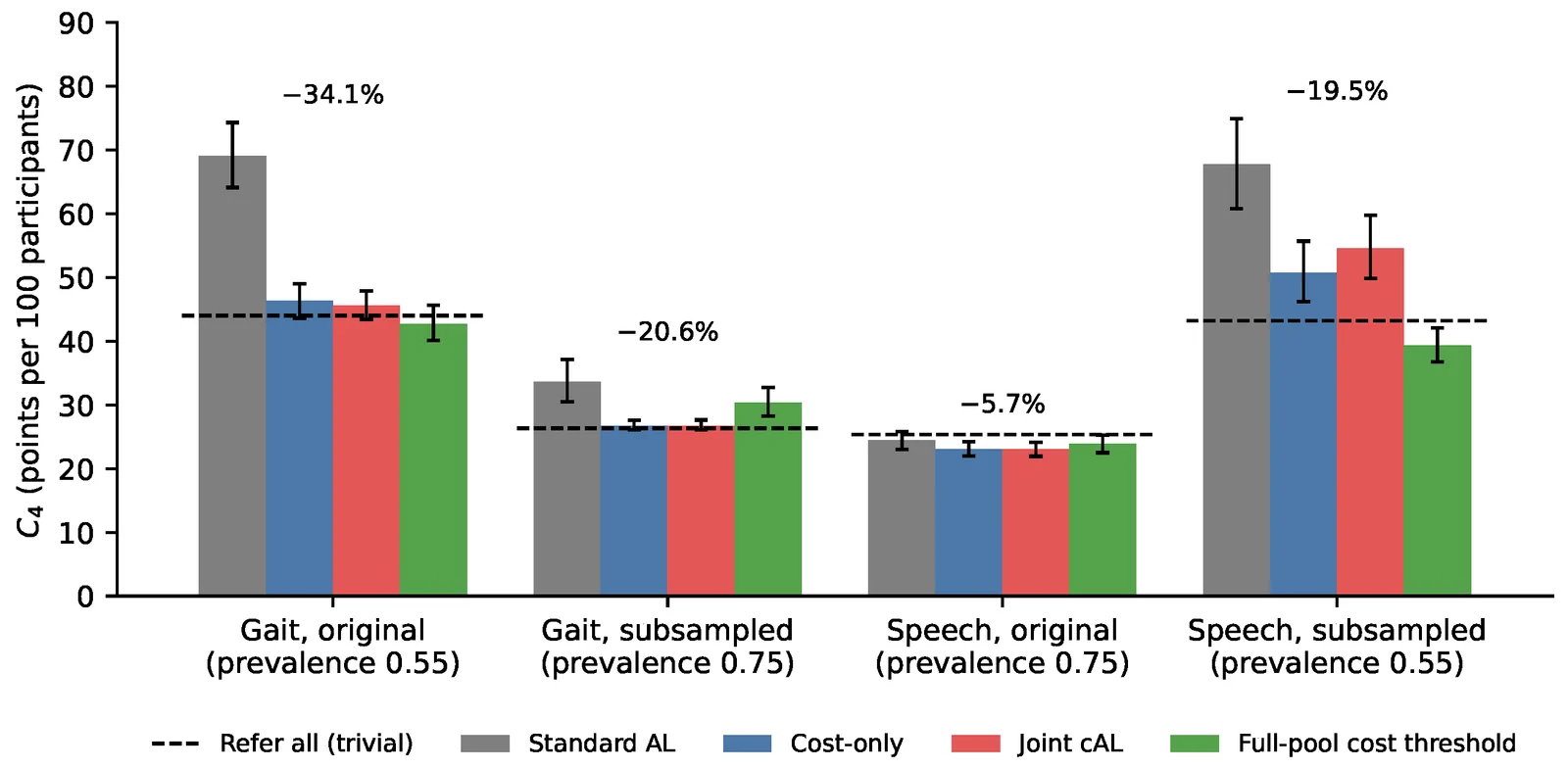
Prevalence-swap experiment. $C_{4}$ at 50% labeling (full-pool cost threshold at 100%) with descriptive bootstrap intervals over 60 splits. Dashed lines mark the refer-all reference, and percentages give the joint-cAL reduction relative to Standard AL.

The prevalence-swap experiment supports a substantial contribution of class balance to the difference in cost reduction between modalities (Figure 8). When gait was subsampled to the speech prevalence of 0.75, the joint-cAL reduction relative to Standard AL fell from 34.1% to 20.6%. When speech was subsampled to the gait prevalence of 0.56, it rose from 5.7% to 19.5%. The mechanism is the position of the symmetric model relative to the refer-all reference. The 4:1 loss refers anyone whose probability of PD exceeds 0.2. At a prevalence of 0.75, a symmetric SVM already predicts PD for almost everyone, and its cost was 0.91 points below the reference. At a prevalence of 0.56, it predicts many negatives and was 25.1 points above the reference. Weighting can recover much of this gap by changing the operating point. However, subsampling also changes cohort size and composition, so this experiment does not isolate prevalence from all other causes or exclude an effect of signal representation. Joint cAL fell clearly below the refer-all reference only in the original speech data (23.02 vs. 25.33). It was slightly above the reference, within its interval, for original gait (45.57 vs. 44.00) and for subsampled gait (26.7 vs. 26.3). It was well above the reference for subsampled speech (54.6 vs. 43.2), where only the full-pool cost threshold went below the reference (39.3).

### 2.6. Cross-Cohort Robustness

Table 6 reports the leave-one-study-out gait results and the speech replication. Cost-sensitive training lowered $C_{4}$ relative to symmetric training in every held-out gait study, and the reduction was largest under the strongest shift. When the treadmill study (Si) was held out, the symmetric models missed more than half of the PD cases (FNR 0.53) and reached $C_{4}$ values above 125. Risk-aware querying added to weighting in Ju and Si (44.9 vs. 46.8 and 58.4 vs. 62.1) but not in Ga (39.7 vs. 39.5). Joint cAL was above the reference in Ga (39.7 vs. 38.3), slightly below it in Ju (44.9 vs. 46.3) and well above it in Si (58.4 vs. 45.3). The cross-validated cost threshold was below the reference in Ga (35.6) but not in Si (47.8). Among the adaptive policies, ClinRisk-LLM had the lowest held-out cost in Ga (39.5), the second lowest in Si (63.5, behind fixed cAL at 53.9) and the highest of the three cost-sensitive policies in Ju (49.7 vs. 46.3–47.7). On the independent speech cohort, as in the Sakar data, the high prevalence left little room for improvement: cost-only and joint cAL were within 0.5 points of the refer-all reference.

### 2.7. Sensitivity to the Batch-Diversity Procedure

Replacing rank spreading with the kernel *k*-means stage of the component ablation changed the gait policy costs by up to 5.7 points (Table 7). Fixed cAL had the lowest gait cost under both procedures (44.47 with *k*-means). The order of the other two reversed: with *k*-means, Adaptive Rule (46.07) was below ClinRisk-LLM (48.60), whereas under rank spreading, ClinRisk-LLM (47.60) was below Adaptive Rule (48.03). For speech, fixed cAL was lowest under both procedures, and the other three policies were within 0.5 points of each other. The cost-sensitive policies thus remained far below Standard AL for gait under either procedure. However, the ranking among them, including the position of ClinRisk-LLM, depended on the diversity procedure. Differences of 2–4 points between these policies should therefore be interpreted together with paired split variability and the sensitivity to batch diversity.

### 2.8. Stability of the Out-of-Fold Calibration Signals

**Figure 9:**
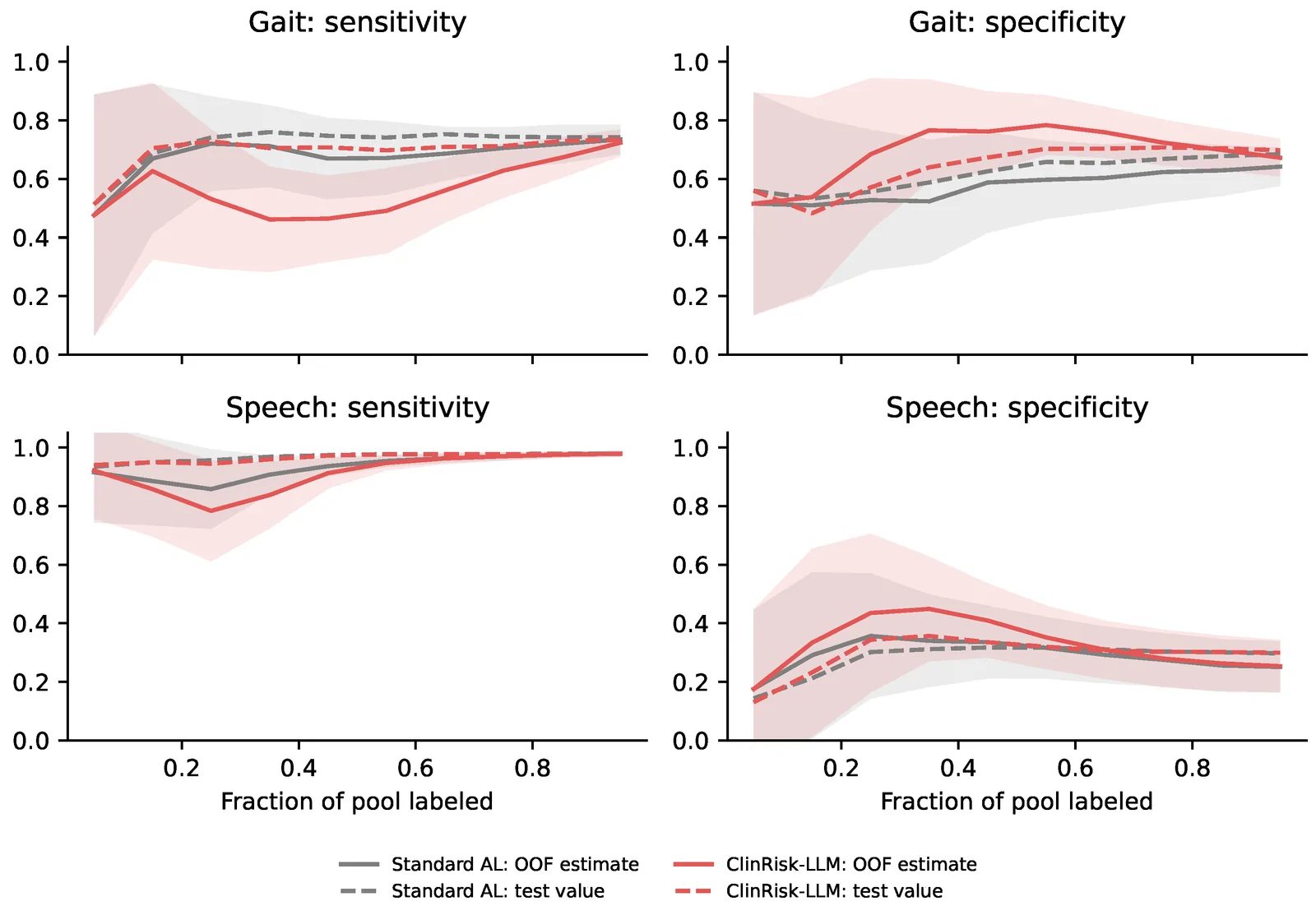
Three-fold OOF estimates of sensitivity and specificity (solid, mean ±1 split-level SD) and the test-set values of the same symmetric SVM (dashed) across the annotation budget, under Standard AL and ClinRisk-LLM acquisition. Gray denotes Standard AL and red denotes ClinRisk-LLM; shaded bands show split-level standard deviation. Test-set values were recorded for this diagnostic only and were never used by the policy.

The OOF signals were unstable early in acquisition and biased by the acquisition rule (Figure 9). In the first 10% of the budget, the cold-start condition applied in 15% of gait steps and 36% of speech steps. Where gait estimates were available, their SD across available observations within the early budget bin was about 0.40, so the early policy decisions were sensitive to small-sample variation. Under Standard AL, the OOF estimates tracked the test-set values with little bias. By 40–50% labeling, their mean absolute error was 0.14 for gait sensitivity and 0.16 for gait specificity. Under ClinRisk-LLM, negative-side querying enriched the labeled set with hard PD cases. As a result, OOF sensitivity underestimated test sensitivity by up to 0.24 and OOF specificity overestimated it by about 0.09–0.13 in the middle of the gait trajectory. This combination is the one that triggers escalation of the risk weight. Under ClinRisk-LLM, decisions at the 0.50 specificity guard agreed with the corresponding test-set decision in 84–97% of gait steps after 30% labeling. Decisions at the 0.70 escalation threshold agreed in only 54–61% of the steps. For speech, the bias was smaller and had largely disappeared by 60% labeling. The OOF signals showed greater agreement for the coarse specificity threshold than for the escalation threshold in these data. This does not validate a clinical safety guard: agreement concerns the symmetric surrogate, can depend on the frequency of each threshold event, and does not guarantee specificity of the weighted classifier. Fine threshold decisions remain unreliable in small, case-enriched gait pools. This helps to explain why policies that differ only in such thresholds diverged after regeneration (Section 2.10).

### 2.9. High-Dimensional Speech Predictors

After unsupervised PCA (mean 61 components), the speech arms gave $C_{4}$ values of 24.31 (Standard AL), 23.98 (query-only), 24.13 (cost-only) and 24.18 (joint cAL) at 50% labeling. ROC-AUC was 0.785 for Standard AL, compared with 0.805 with the raw predictors. Discrimination was not improved by this particular dimensionality reduction. This sensitivity analysis does not establish the absence of high-dimensional overfitting or exclude a benefit from other regularization strategies. The PCA models predicted PD for almost every participant, with specificity of 0.09–0.12, and so lay just below the refer-all reference (25.33). With no room left for weighting to act, the four arms became indistinguishable. This is consistent with a substantial influence of class balance on the speech operating point, but does not separate that influence from feature representation or sample size.

### 2.10. Sensitivity of ClinRisk-LLM to Policy Regeneration

All 40 regenerated policies (20 from Claude Sonnet 5 and 10 each from Claude Opus 5.5 and Claude Haiku 5.5) passed the static checks and ran without failure on every split. Their behavior differed markedly from the original frozen policy (Table 8). For speech, every regenerated policy kept training symmetric ($r_{t}=1$ throughout) and reached $C_{4}$ between 24.51 and 24.93, consistent with the original policy (24.69). For gait, the regenerated policies were consistently more conservative. Their mean risk weight was 1.66 (range 1.18–2.58), compared with 3.47 for the original. Mean $C_{4}$ was 67.27 (SD 5.10; range 55.90–75.83). This is much closer to Standard AL (72.77) than to the original policy (47.60), and no Sonnet 5 regeneration came within 8 points of the original. The original gait result was therefore not reproduced by regenerating the policy from the archived specification. The original user prompt was subsequently recovered from the repository generation archive and differs from the reconstruction used for these 40 generations. These results therefore do not isolate repeated sampling under identical original wording; they demonstrate variation under the reconstructed specification and a gap from the original frozen implementation. The lower risk weights are consistent with a stricter reading of the clause “protect specificity first”, with higher thresholds before sensitivity pressure is escalated; the regenerated policies and their results are archived in the repository. The alternative models gave the same picture. For gait, Opus 5.5 policies were less conservative (mean $r_{t}$ 2.60) and reached 55.33 (range 52.10–57.00), whereas Haiku 5.5 policies reached 65.99 (63.07–70.00). Neither model reproduced the original 47.60; the closest regeneration was 4.5 points higher. For speech, all three models kept training symmetric, with costs between 24.49 and 25.04. Thus, the choice of LLM and the particular generation changed the gait cost by more than 20 points (52.10–75.83). In the internal evaluation, no LLM-derived policy performed consistently better than the simple fixed or deterministic policies. In the external gait cohorts, the original policy had the lowest cost among the adaptive policies in one held-out study, the second lowest in another and the highest among the cost-sensitive policies in the third (Section 2.6).

## 3. Discussion

### 3.1. Principal Findings

Cost sensitivity reduced the prespecified asymmetric loss, most clearly in gait. With half of the pool labeled, joint cAL lowered $C_{4}$ by 34.1% for gait and 5.7% for speech relative to Standard AL. The ablations attribute most of this reduction to classifier weighting. Risk-aware querying added a small mean gain for gait that was not consistent across paired splits, and a 0.3% mean reduction for speech. A symmetric model with a threshold tuned on the labeled set came within 1.3 points of the joint-cAL gait cost. Even so, the early plateaus suggest that the chosen operating points can often be reached without labeling the entire pool.

These relative reductions need a reference point. The refer-all policy provides an essential decision-analytic reference under asymmetric loss. Joint cAL brings gait cost close to this reference (45.57 vs. 44.00), compared with 69.10 for Standard AL, while speech joint cAL improves on its corresponding reference (23.02 vs. 25.33). These comparisons distinguish improvement over the learned symmetric operating point from improvement over an unconditional referral strategy. The prevalence-swap experiment supports class balance as an important contributor to the gait–speech contrast, although sample size and composition changed simultaneously. When the prevalences were exchanged, the two modalities gave almost identical reductions (20.6% and 19.5%). The larger gait gain should therefore not be read as evidence that gait carries more disease-relevant information than speech.

ClinRisk-LLM translated the safety specification into an auditable policy and adjusted risk settings to each modality: relatively high weights for gait and near-symmetric weighting for speech. The controlled comparisons distinguish this operational contribution from the cost reductions associated with weighting. In the adaptive comparison, fixed cAL has the lowest mean cost in both modalities; ClinRisk-LLM has lower gait cost than Adaptive Rule in 52% of splits and than fixed cAL in 43%, with rankings affected by batch diversity. The 40-policy analysis further shows stable near-symmetric speech behavior and a wider range of gait operating points under the reconstructed specification. That specification differs from the recovered original wording, so its comparison with the original does not isolate sampling variability. Together, these findings support reporting the prompt, generated rules and executable policy as separate artifacts, and evaluating several generated policies before selecting a policy for a new study. The exact original prompt and response are archived in the repository generation archive.

These findings complement retrospective PD classification studies by evaluating the decision consequences of the operating point alongside case–control discrimination [16,17]. In our setting, the cost-sensitive gains arose mainly from moving the operating point, while ROC-AUC under symmetric scoring remained essentially unchanged. Reporting the full error trade-off, rather than a single accuracy figure, is therefore necessary whenever an AI model is tuned to reduce missed cases.

The lower FNR came with lower specificity, particularly for gait. The framework therefore changes the balance between errors rather than improving every aspect of performance. The 4:1 loss is an experimental preference, not an established clinical utility. Gait and speech also differ in sample size, class balance and feature space. Their results should not be used to claim that one modality is intrinsically better.

### 3.2. Relation to Threshold Adaptation and Cost-Sensitive Active Learning

Our results connect to two lines of work. In threshold adaptation, the decision threshold of an existing model is moved to reflect class imbalance or unequal error costs, without retraining. GHOST, for example, selects the threshold from out-of-sample predictions and improves minority-class performance across many imbalanced datasets [25]. Work on clinical prediction models stresses that such thresholds should follow the clinical consequences of errors and that predicted risks must be calibrated before they are compared with a threshold [26,27]. Our threshold-tuned reference recovered most of the benefit of class-weighted active learning in gait. This suggests that post hoc thresholding should be reported as a standard comparator for cost-sensitive active learning. In active learning, the methodological literature has moved toward deep, batch-mode and diversity-aware acquisition [28], and medical applications increasingly report the annotation savings of such methods [29]. Cost-sensitive variants weight either the cost of labeling or the cost of misclassification [18,19]. Cost-sensitive acquisition has also been studied directly: COAL estimates label-specific costs and queries potentially optimal labels [30], rather than merely rescaling a binary SVM margin. Recent batch-AL theory explicitly balances informativeness and diversity [31]. These approaches clarify why acquisition, training loss and threshold selection are distinct design choices. The present experiment does not benchmark COAL or establish guarantees comparable to that theory; its contribution is an empirical ablation in small PD biosignal cohorts. The common asymmetric loss, refer-all reference and paired split-level comparisons used here make the separate effects of these design choices explicit. In our small tabular biosignal cohorts, the acquisition rule mattered much less than the operating point.

### 3.3. Is the Loss of Specificity Acceptable?

Whether the lower specificity is acceptable depends on the setting and on the confirmatory test. In a hypothetical specialist-referral population with 30% prevalence, joint cAL needed about 6.2 extra total confirmatory tests for each additional case detected, including 5.2 unnecessary tests. At the prespecified 4:1 loss, its net benefit is 0.148 compared with 0.166 for Standard AL; the pairwise break-even exchange rate is approximately 5.24:1. These quantities specify the utility preference needed to justify the sensitivity gain in this scenario. The burden must be assessed for the particular confirmatory procedure, its harms, capacity and costs. These scenarios transport clinical PD sensitivity and specificity; they do not estimate performance against molecular positivity. For speech, at the same 30% prevalence, joint cAL would detect about three additional cases per 1000 people for about 53 additional total tests (roughly 16.5 tests per additional case). This is because the symmetric speech model is already highly sensitive. In population screening, the same gait operating point would send nearly four-fifths of all people to confirmatory testing, at a PPV near 1%, and its net benefit would be negative. The decision curves add a further point. The full-pool symmetric model has net benefit close to refer-all at the 4:1 exchange rate, with greater separation at thresholds that assign more weight to unnecessary tests. The cost ratio therefore determines how discrimination translates into decision utility. Test-specific utility elicitation and a representative target population are needed to select an operating point for a confirmatory pathway.

### 3.4. Prospective Relevance to Biological and Molecular Characterization of PD

This section describes a prospective application that the present data cannot test. The calibration loop is not specific to a signal type or classifier. Its three components, the labeled-pool context vector, the frozen calibration policy and the risk-adjusted query score, require only a decision function and an oracle that returns a label at a cost. In this study, the oracle was a clinical diagnosis, and no biological or molecular measurement was available. In principle, the same loop could be applied when the label comes from a costly biological measurement, such as an α-synuclein SAA, dopamine transporter imaging or genetic testing [12,13]. The acquisition rule would then determine which individuals are sent for confirmation, and the asymmetric cost would encode the consequence of missing a person with underlying synucleinopathy. Biological staging frameworks define categories that may be heterogeneous in their motor and speech presentation [10,11]. Whether inexpensive digital phenotypes can be related to these molecularly defined groups with fewer assays is an open question. Our results do not show that gait or speech signals identify biological PD or stage. The refer-all and decision-curve analyses identify the utility and prevalence conditions that such a confirmatory pathway would need to address. Testing this application requires cohorts in which digital recordings and biological measurements are collected from the same participants, with costs that reflect the specific confirmatory test.

The SVM was chosen to keep the ablation transparent and computationally stable at small sample sizes. Deep neural networks, including models that learn directly from raw gait force signals or speech spectrograms, can be used in the same loop if they provide a margin or a calibrated probability. In that case, the class weight becomes a weighted loss and the query score is computed from the network output. Because the LLM produces a frozen policy rather than acting on individual participants, replacing the classifier does not change the audit trail of the calibration step.

### 3.5. Limitations and Future Work

The methodological findings define several priorities for further validation. All datasets are retrospective and case-enriched. External validation was limited to held-out studies within one gait database and to a small independent speech cohort with incompatible features, so no fitted model was transferred across speech cohorts. The held-out treadmill study showed that site and protocol shifts can push every model above the refer-all reference. The original user prompt, invocation command and returned policy are archived in the repository generation archive. Policy generation used Claude Code CLI v2.1.241 with its default system configuration; no custom system-prompt override was supplied. The regeneration analysis used a reconstruction prepared before that recovery; it therefore does not isolate variability under the original wording. Cross-modal fusion could not be tested, because no participant contributed both gait and speech. Cohorts with paired recordings, such as smartphone-based studies that collect walking and voice tasks from the same individuals, are needed to test whether jointly calibrated modalities lower the cost further. Independent clinical assessment remains a further validation step. In future work, we plan a structured rule-by-rule assessment of the safety specification, numerical thresholds and frozen and regenerated policies by independent movement-disorder specialists. This assessment will examine clinical plausibility, missing safeguards and suitability for specific referral settings, with individual judgments and disagreements documented. The OOF signals that drive the policy were noisy early in acquisition and biased by the acquisition rule, which limits fine-grained adaptive calibration in small pools. ClinRisk-LLM should therefore be understood as a population-level learning strategy, not as a clinical diagnostic system. Further work needs prevalence-representative cohorts, costs elicited from clinical practice and policies that reduce missed cases without allowing for specificity to collapse. Multimodal cohorts that pair digital gait and speech recordings with SAA, imaging and genetic data would allow for the framework to be evaluated against biological rather than purely clinical labels.

## 4. Materials and Methods

This section first defines the learning framework and the ClinRisk-LLM policy (Section 4.1, Section 4.2 and Section 4.3) and then describes the datasets, baselines and evaluation protocol (Section 4.4, Section 4.5, Section 4.6, Section 4.7 and Section 4.8). The framework has three separate functions. Acquisition chooses the observations to label. Cost-sensitive training controls their influence on the classifier. Adaptive calibration sets the risk level at each budget step. ClinRisk-LLM transfers this calibration principle from remote-sensing anomalies to participant-level PD biosignals [24]. We define these functions before presenting the ClinRisk-LLM policy and the full iterative procedure.

All analyses were conducted in Python 3.9.6 using scikit-learn 1.6.1, NumPy 2.0.2, SciPy 1.13.1, pandas 2.3.3, and Matplotlib 3.9.4. Policy generation used Claude Code CLI v2.1.241, as described below.

### 4.1. Problem Formulation

Let $X\subset R^{p}$ denote the participant-level feature space and Y={0,1} the label space, where 1 denotes PD. For each outer split, the available development data form a pool $U_{0}={\left\{x_{i}\right\}}_{i=1}^{n}$ and an independent test set is held aside. AL starts from a label-blind initial sample $L_{0}$ and, at iteration t=1,…,T, selects a batch $Q_{t}\subset U_{t-1}$ for oracle annotation:

(1) $$L_{t}=L_{t-1}\cup \left\{\left(x,O\left(x\right)\right):x\in Q_{t}\right\},\;U_{t}=U_{t-1}\setminus Q_{t}.$$

For a labeling budget *B*, the operational objective is to minimize a prespecified asymmetric population risk while retaining the full error trade-off:

(2) $$\min_{Q_{1},\ldots ,Q_{T},f_{T}}R_{4}\left(f_{T}\right),\;\left|L_{T}\right|\leq B,$$

where

(3) $$R_{4}\left(f\right)=4\;\mathrm{Pr}\left\{f\left(X\right)=0,Y=1\right\}+\mathrm{Pr}\left\{f\left(X\right)=1,Y=0\right\}.$$

Its finite-test-set estimate, $C_{4}$, is defined in Section 4.8. The ratio four is an experimental operating preference and is not asserted to be a clinically established utility.

### 4.2. Classifier and Query Rules

#### 4.2.1. Cost-Sensitive SVM

All classifiers were RBF support vector machines with C=1. Symmetric training used $w_{0}=w_{1}=1$. Cost-sensitive training used $w_{0}=1,w_{1}=4$, with PD as class 1. Class weighting changes the fitted support vectors and coefficients and was not assumed equivalent to applying a fixed analytic threshold to a symmetric SVM.

For the SVM objective, the binary labels are recoded as ${\tilde{y}}_{i}=2y_{i}-1\in \left\{-1,+1\right\}$. Using θ for the separating hyperplane, β for its intercept and $\omega_{y_{i}}$ for the class weight, the weighted soft-margin problem is

(4) $$\min_{\theta ,\beta ,\xi }\frac{1}{2}{∥\theta ∥}^{2}+C\sum_{i=1}^{|L_{t}|}\omega_{y_{i}}\xi_{i},\;{\tilde{y}}_{i}\left\{\theta^{⊤}\phi \left(x_{i}\right)+\beta \right\}\geq 1-\xi_{i},\;\xi_{i}\geq 0.$$

Unlike a post hoc threshold change, weighting can change the support vectors and the fitted decision function itself.

#### 4.2.2. Risk-Sensitive Uncertainty and Diversity

Let d(x) denote the SVM decision function, with d(x)>0 assigned to PD and d(x)≤0 assigned to control. Standard uncertainty ranked observations by |d(x)|. Risk-sensitive querying used

(5) $$q\left(x\right)=\left\{\begin{array}{cc} |d(x)|/4, & d(x)\leq 0,\\ |d(x)|, & d(x)>0, \end{array}\right.$$

and selected the lowest scores. Thus, currently negative observations, the region containing possible FNs, received greater priority. The 25 lowest-score candidates were divided into five clusters by RBF-kernel *k*-means with ten random restarts. The lowest-score member of each cluster was queried.

#### 4.2.3. Formal Active-Learning Procedure

At each budget step, the current SVM was fitted on $L_{t}$. Decision scores were then computed for $U_{t}$. Random selection or the specified uncertainty rule generated 25 candidates. Kernel *k*-means supplied batch diversity and five observations were labeled. The classifier was then refitted. Test data were used only for evaluation and never influenced acquisition.

### 4.3. ClinRisk-LLM Adaptive Calibration

The policy was generated on 23 August 2026 with Claude Sonnet 5 through the Claude Code CLI. The model received only the allowed context variables, the discrete action space and the instruction to protect specificity before increasing sensitivity pressure. It produced a structured policy that was frozen before evaluation. At iteration *t*, the context vector was

(6) $$c_{t}=\left(b_{t},{\hat{\mathrm{Sens}}}_{t},{\hat{\mathrm{Spec}}}_{t},\pi_{t},m_{t},{\bar{p}}_{t}^{-},g_{t}\right),$$

Here, $b_{t}=\left|L|/\right|U_{0}|$ is the fraction of the pool already labeled. Sensitivity and specificity are three-fold out-of-fold estimates from a symmetric SVM fitted within the labeled set. The term $\pi_{t}$ denotes labeled PD prevalence. For OOF monitoring, the SVM bandwidth is recomputed on the currently labeled subset; the main weighted classifier uses the development-pool bandwidth. Both exclude test data. The median unlabeled absolute margin under the previous risk setting is $m_{t}={\mathrm{median}}_{x\in U}\left|{\bar{d}}_{t}\left(x\right)\right|$. The corresponding negative-prediction fraction is ${\bar{p}}_{t}^{-}={\left|U\right|}^{-1}\sum_{x\in U}I\left\{{\bar{d}}_{t}\left(x\right)\leq 0\right\}$. Finally, $g_{t}\in \left\{\mathrm{declining},\mathrm{flat},\mathrm{improving}\right\}$ records the recent OOF sensitivity trend. When either labeled class contains fewer than three observations, the OOF quantities are treated as unavailable and the policy uses its prespecified cold-start action. Test statistics are excluded. OOF estimates were chosen as an internal performance signal that reuses the acquired labels without reserving a separate validation sample. Cross-fitting reduces direct training-set optimism, but does not correct acquisition-induced selection bias. A random audit sample or a separate representative validation set would provide a different monitoring design at an additional labeling cost. However, the OOF estimates are computed from few observations that active learning has selected non-randomly. Their stability and bias were therefore examined directly (Section 2.8). At every step, the OOF estimates were compared with the test-set sensitivity and specificity of the same symmetric SVM. These test-set values were recorded for this diagnostic only and were never passed to the policy.

The policy chooses risk weight $r_{t}$ and negative-side query priority $a_{t}$ from {1,2,3,4}. If the OOF quantities are unavailable, it targets (2,2). Specificity below 0.50 activates the emergency guard $\left(r_{t},a_{t}\right)=\left(1,4\right)$. Otherwise, specificity below 0.60 caps $r_{t}$ at 2 and targets $a_{t}=3$. With specificity at least 0.70, sensitivity below 0.60 targets $r_{t}=4$, and sensitivity below 0.75 targets $r_{t}=3$. The latter condition also targets $a_{t}=3$ when the negative-prediction fraction is at least 0.85. A declining sensitivity trend with specificity at least 0.70 and $m_{t}\geq 0.10$ raises $r_{t}$ by one. When $m_{t}<0.10$ and specificity is at least 0.60, $a_{t}$ is raised to at least 3. An observed labeled prevalence outside [0.15,0.85] raises it to at least 2. Except for the emergency guard, both actions can change by at most one level per iteration. The risk weight is held at its preceding value once 85% of the pool has been labeled. These rules and thresholds were fixed before evaluation. The four design principles address asymmetric errors, specificity loss, unstable feedback and late-budget oscillation. They are methodological safeguards rather than clinically established requirements. The numerical cutoffs in the frozen policy were proposed in the recovered LLM response and adopted as implementation choices and have not been validated as clinical safety limits; the 4:1 loss is likewise an experimental preference. The archived repository documents the design rationale, frozen policy parameters and their provenance. In particular, the specificity guard uses OOF estimates from a symmetric surrogate model, not the deployed weighted classifier, and therefore does not guarantee a minimum test-set specificity.

The safety specification has four clauses: (i) missed PD cases are costlier than false alarms, but specificity is protected before sensitivity pressure is increased; (ii) sensitivity pressure is escalated only when specificity has headroom and sensitivity is poor or declining; (iii) if specificity collapses, the risk weight is reduced at once and targeted querying of possible false negatives is used instead; and (iv) actions change gradually and the risk weight is frozen late in the budget. The model was restricted to the seven context variables above and to the action space ${\left\{1,2,3,4\right\}}^{2}$. The authors checked the returned rules for consistency with these clauses and for test independence before freezing them. The original experiment-specific user prompt and returned JSON were recovered on 18 October 2026 from the contemporaneous execution transcript and are available with timestamps and checksums in the repository generation archive. The original prompt specifies the allowed inputs, actions, asymmetric loss, exclusion of test signals and conservative escalation/de-escalation goals. Numerical cutoffs, defaults, smoothing and the late-budget freeze appear in the returned JSON and were transcribed into the frozen Python 3.9.6 implementation. The four clauses above summarize that design; the later reconstruction used for the revision generations is also archived in the repository. The successful historical command used Claude Code CLI 2.1.241 without explicit model or system-prompt flags; the contemporaneous CLI banner reported Sonnet 5. Policy generation used Claude Code CLI v2.1.241 with its default system configuration; no custom system-prompt override was supplied. The archived repository documents the experiment-specific instructions and the resulting policy used in the evaluation. The Python implementation gives the emergency guard precedence over the late-budget freeze; both the raw JSON and executed code are archived to expose this precedence choice.

LLM output can vary between generations. To characterize policy variation under the documented specification, the archived specification was submitted 20 times, in independent sessions, to the same model (Claude Sonnet 5, Claude Code CLI). Each response had to be a pure Python function of the allowed inputs. Responses were stored verbatim and statically checked: imports, input/output and attribute access other than dictionary look-up were rejected, and actions outside {1,2,3,4} were treated as failures. Helper functions defined within the returned code were permitted. To assess alternative LLM configurations, the same specification was also submitted 10 times each to Claude Opus 5.5 and Claude Haiku 5.5. Every valid policy was then replayed without modification through the same adaptive loop on all 60 splits.

The independently specified Adaptive Rule uses different thresholds. Specificity below 0.50 selects (1,3). Sensitivity below 0.65 with specificity at least 0.70 targets (4,3). Sensitivity below 0.80 with specificity at least 0.65 targets (3,2). Otherwise, the rule returns toward (2,2). Standard AL fixes (r,a)=(1,1) and fixed cAL uses (4,4).

**Figure 10:**
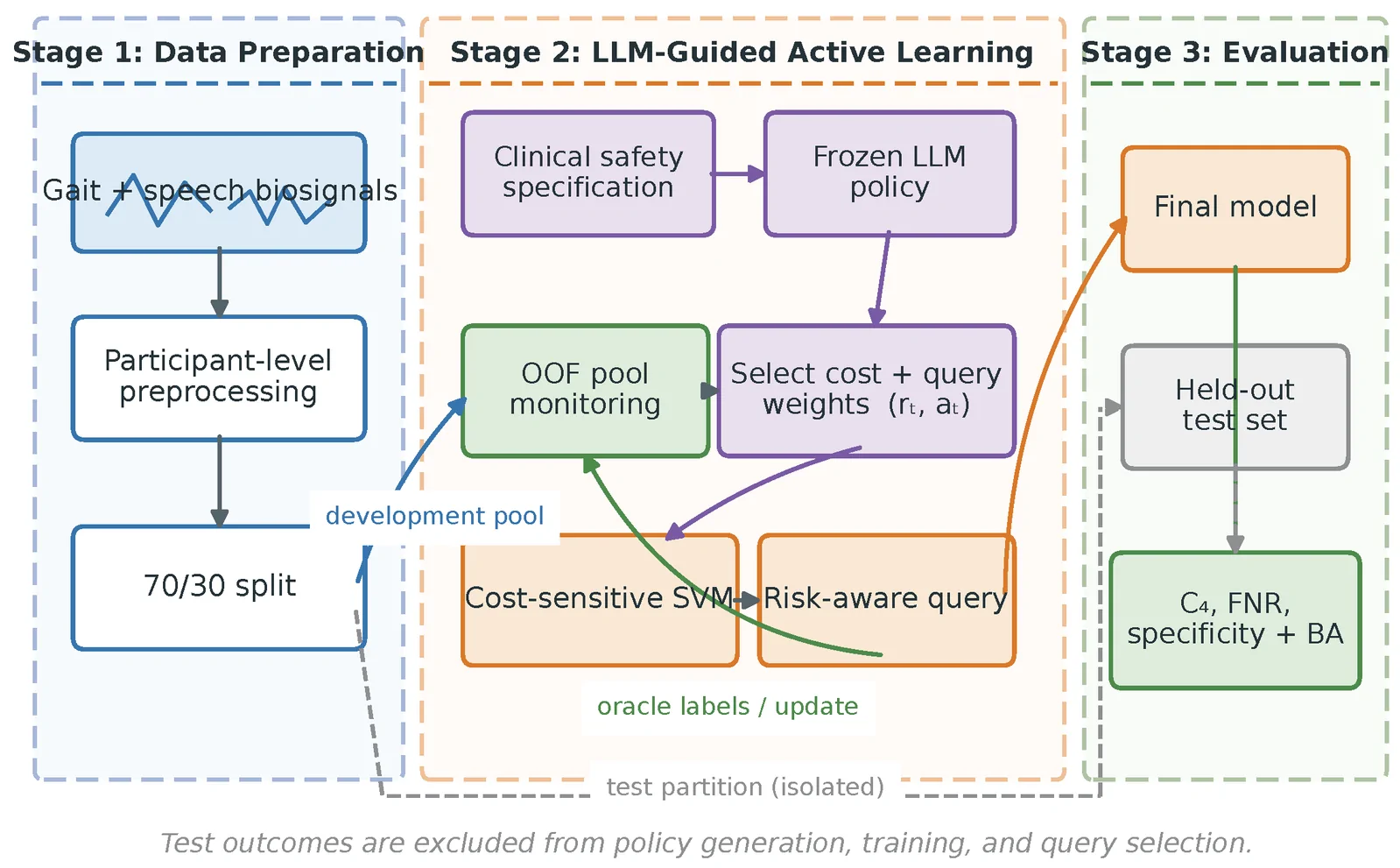
Architecture of the proposed LLM-guided adaptive cost-learning framework.

For the four-policy adaptive comparison, we ordered the 25 observations with the lowest risk-adjusted scores. We then selected five approximately equally spaced positions from this ordered candidate set. This deterministic rank-spreading step prevents a batch from containing only near-duplicate margin positions. It also keeps the adaptive comparison computationally identical across policies. The procedure differs from the kernel *k*-means diversity stage in the component-wise cAL ablation described in Section 4.2.2. The adaptive-policy costs are therefore not directly comparable with the component-ablation costs reported in the Section 2. There are also implementation differences: the component analysis truncates the last batch to the requested label count, whereas the adaptive loop advances in complete batches and records a nominal budget when the labeled fraction is within one percentage point below it or has passed it. At the nominal 50% point, the component targets are 58 gait and 88 speech labels; the adaptive records contain 60–61 gait and 87–91 speech labels. Kernel *k*-means restarts also use different random-number schedules in the two loops. The alternative-diversity experiment isolates the diversity change within the adaptive loop; it does not make that loop identical to the component analysis. Policy effects should therefore be interpreted within each comparison. To check that the policy comparison does not depend on this choice, the four policies were also rerun with the kernel *k*-means stage (Section 2.7). Figure 10 summarizes the complete information flow from data preparation to final evaluation.

As shown in Figure 10, data preparation, adaptive learning and evaluation follow separate paths. The frozen policy selects $\left(r_{t},a_{t}\right)$ using development-pool signals only. These values determine the weighted classifier and the acquisition rule used to request the next labels. The held-out test partition enters the workflow only after the final model has been fixed.

#### ClinRisk-LLM: Formal Procedure

Algorithm 1 gives the complete adaptive calibration loop. The policy generator is invoked once before resampling. No language-model call, test outcome or participant-level recommendation occurs inside the loop.
**Algorithm 1** ClinRisk-LLM Adaptive Cost Calibration.***Require:*** 
*Development pool $U_{0}$, label-blind initial set $L_{0}$, and annotation budget B****Require:*** 
*Batch size b=5, candidate count K=25, oracle O, and frozen policy P****Ensure:*** 
*Final classifier $f_{T}$ and risk trajectory ${\left\{\left(r_{t},a_{t}\right)\right\}}_{t=1}^{T}$**  1:* 
*$L\leftarrow L_{0}$; $U\leftarrow U_{0}\setminus \left\{x:\left(x,y\right)\in L_{0}\right\}$; $\left(r_{0},a_{0}\right)\leftarrow \left(1,1\right)$; H←[]; t←0**  2:* 
***while*** *|L|<B and U≠⌀* ***do***
*  3:* 
*   t←t+1**  4:* 
*   Estimate three-fold OOF sensitivity and specificity using L only**  5:* 
*   Fit a provisional SVM using $r_{t-1}$ and compute margins ${\bar{d}}_{t}\left(x\right)$ for x∈U**  6:* 
*   Form $c_{t}=\left(b_{t},{\hat{\mathrm{Sens}}}_{t},{\hat{\mathrm{Spec}}}_{t},\pi_{t},m_{t},{\bar{p}}_{t}^{-},g_{t}\right)$**  7:* 
*   $\left(r_{t},a_{t}\right)\leftarrow P\left(c_{t},r_{t-1},a_{t-1}\right)$ {specificity guard, one-level smoothing, late-budget freeze}**  8:* 
*   Fit $f_{t}$ on L with class weights $\left(\omega_{0},\omega_{1}\right)=\left(1,r_{t}\right)$ and set $d_{t}\left(x\right)\leftarrow f_{t}\left(x\right)$ for x∈U**  9:* 
*   $q_{t}\left(x\right)\leftarrow \left|d_{t}\left(x\right)\right|/a_{t}$ if $d_{t}\left(x\right)\leq 0$, and $q_{t}\left(x\right)\leftarrow \left|d_{t}\left(x\right)\right|$ otherwise**10:* 
*   $S_{t}\leftarrow$ the min(K,|U|) observations with the smallest $q_{t}\left(x\right)$**11:* 
*   $Q_{t}\leftarrow$$\min(b,|S_{t}|,B-|L|)$ approximately equally spaced ranks from ordered $S_{t}$**12:* 
*   $L\leftarrow L\cup \{\left(x,O\left(x\right)\right):x\in Q_{t}\}$; $U\leftarrow U\setminus Q_{t}$**13:* 
*   Append $\left(c_{t},r_{t},a_{t}\right)$ to H**14:* 
***end** **while***
*15:* 
*Refit $f_{T}$ on the final labeled set using the last selected $r_{t}$**16:* 
***return*** *$f_{T},{\left\{\left(r_{t},a_{t}\right)\right\}}_{t=1}^{T}$*

### 4.4. Datasets and Representations

We evaluated the framework on one gait dataset and one speech dataset. The following subsections describe how participant-level observations were constructed, how leakage was avoided, and how the comparison arms were run and evaluated.

#### 4.4.1. Gait: PhysioNet Vertical Ground-Reaction Force

The gait analysis used the PhysioNet Gait in Parkinson’s Disease Database [20,21]. The demographics file lists 166 participants. For each participant, the first recorded walk was processed using the total vertical force under each foot. Heel strikes and toe-offs were detected as crossings of a 20 N contact threshold. Stride, swing and stance intervals outside plausible bounds (0.6–2.5 s, 0.2–1.2 s and 0.3–1.8 s, respectively) were discarded as artifacts, and at least five valid strides per foot were required. The walk was then summarized by 12 prespecified subject-level features: cadence, bilateral swing-time asymmetry, left/right mean stride, swing and stance times, and left/right coefficients of variation for stride and swing time. No recording was excluded by these quality rules. One control listed in the demographics file has no recording in the database. All files were verified against the published SHA-256 checksums. The final cohort comprised 165 participants (93 PD, 72 controls) with no missing feature values. Splits were participant-level. The database pools three independently conducted studies with different protocols: a dual-task study (Ga; 29 PD, 18 controls) [32], a rhythmic auditory stimulation study (Ju; 29 PD, 25 controls) [33] and a treadmill study (Si; 35 PD, 29 controls) [34]. The first recorded walk of each participant was used in all three studies. This structure allows for each study to be held out as an external cohort (Section 4.11).

#### 4.4.2. Speech: Patient-Aggregated Acoustic Predictors

The speech analysis used the Parkinson’s Disease Classification dataset [22,23]. Each participant contributed exactly three recordings, which were averaged feature by feature. This produced 252 participant observations, comprising 188 PD cases and 64 controls. The dataset contains no missing values, so no imputation was required. Predictors were standardized using the development pool only, and the RBF bandwidth was set to $1/(p\;\hat{\mathrm{Var}}\left(X\right))$ from the same pool. Removing the identifier and outcome columns left 753 predictors. The complete prespecified predictor set was used. A previously explored seven-feature wrapper-selected subset was excluded. Selection outside the outer resampling loop would leak held-out outcomes. A previously explored gait autoencoder was also excluded. Its fixed pretraining partition was not disjoint from every resampled test set. With 753 predictors, the labeled set is smaller than the number of features at every budget. To check for high-dimensional instability, the speech ablation was repeated after an unsupervised principal component analysis. The analysis was fitted to each development pool and retained 90% of the variance. Table 9 summarizes the resulting participant-level cohorts and makes their structural differences explicit.

#### 4.4.3. Independent Speech Cohort

An independently recorded speech cohort was used for replication: the UCI Parkinsons dataset of sustained-phonation dysphonia measures [35,36]. It contains 195 recordings with 22 features. Parsing the speaker identifiers in the file yields 32 speakers (24 PD), whereas the repository description reports 31 participants (23 PD). Its features are not shared with the Sakar set. A model fitted on one cohort therefore cannot be transferred to the other. Instead, the complete framework was rerun on this cohort. Each recording was an analysis observation, and splits were grouped by speaker identifier. Performance metrics were calculated per recording, not after aggregating predictions by speaker; consequently, speakers with more recordings contribute more to the reported metrics.

The cohorts differ in ways that matter for interpretation (Table 9). Gait has 165 participant-level observations and only 12 interpretable temporal features. The Sakar speech cohort has 252 participant-level observations and 753 acoustic predictors. The UCI replication has 195 recording-level observations from 32 speaker identifiers. We therefore report the modalities separately and do not compare their raw costs as though they came from a common population.

### 4.5. Participant-Level Splitting and Preprocessing

We used 60 repeated class-stratified 70/30 pool/test splits. Repetitions overlap and are not independent population samples. They describe stability to splitting. Standardization and RBF bandwidth were estimated from the pool only. The initial labeled set comprised 10 observations sampled without inspecting labels. If it contained one class, additional random observations were labeled, each counting against the budget, until both classes were represented.

### 4.6. Baselines and Component Ablation

Five arms were evaluated:

1. Random querying with symmetric training.
2. Standard uncertainty–diversity querying with symmetric training.
3. Risk-sensitive querying with symmetric training (query-only).
4. Standard querying with class-weighted training (cost-only).
5. Risk-sensitive querying with class-weighted training (joint cAL).

Full-pool symmetric and class-weighted SVMs were included. A third full-pool reference selected a threshold from five-fold out-of-fold pool scores to minimize the same 4:1 expected cost. A trivial refer-all policy, which labels every participant PD and therefore has FN=0 and FP equal to the number of controls, was added as an unconditional referral reference for evaluating sensitivity-weighted models. Two further arms tested whether post hoc thresholding alone reproduces the gains of cost-sensitive AL at partial budgets. Both used symmetric training with either standard or random acquisition. At each reported budget, a decision threshold was chosen to minimize the 4:1 expected cost, using five-fold out-of-fold scores within the labeled set (fewer folds when a class had fewer than five labels). The test set was not used. The adaptive comparison additionally included Standard AL, fixed cAL, the independently specified Adaptive Rule and ClinRisk-LLM.

### 4.7. Experimental Configuration

To ensure a controlled comparison, the same experimental settings were used for every policy except for the training weights and acquisition rules being evaluated. These shared settings are listed in Table 10.

As listed in Table 10, every arm uses the same split ratio, initialization, batch size, classifier family and evaluation loss. At a given budget, the arms differ only in their training weights and acquisition rules. The 60 repetitions are overlapping partitions of the same datasets. They measure sensitivity to the split and are not independent clinical replications.

### 4.8. Evaluation Metrics and Statistical Reporting

Every method used the common evaluation cost

(7) $$C_{4}=100\frac{4\;\mathrm{FN}+\mathrm{FP}}{N},\;\mathrm{FNR}=\frac{\mathrm{FN}}{\mathrm{TP}+\mathrm{FN}},\;\mathrm{Specificity}=\frac{\mathrm{TN}}{\mathrm{TN}+\mathrm{FP}},$$

where N=TP+TN+FP+FN is the number of test participants. Thus, $C_{4}$ is a weighted cost index expressed as points per 100 participants. Unlike an error percentage, it can exceed 100 when false negatives are frequent. FNR, specificity, precision, balanced accuracy and ROC-AUC were also recorded. We report split-level means and percentile bootstrap intervals as descriptive resampling summaries. Because splits overlap, ordinary Wilcoxon *p*-values were not used and intervals are not interpreted as independent-population confidence intervals. All preprocessing, model fitting and evaluation procedures were defined before the final analysis and applied identically across the compared methods.

### 4.9. Downstream Testing Burden and Decision Curves

The 4:1 loss is equivalent to referring a person for confirmatory testing when the probability of PD exceeds the threshold $p_{t}=1/\left(1+4\right)=0.2$. Net benefit at this threshold [27] is NB(0.2)=(TP−0.25FP)/N. It is linked to the cost index by $C_{4}/100=4\pi -4\;\mathrm{NB}\left(0.2\right)$, where π is the test-set prevalence. $C_{4}$ is thus a rescaled net benefit, and the refer-all policy is the usual reference strategy of decision curve analysis. Decision curves over $p_{t}\in \left[0.05,0.60\right]$ were computed for full-pool symmetric SVMs with Platt-scaled probabilities. To express the burden in clinical units, the mean sensitivity and specificity of each operating point were applied to hypothetical populations of 1000 people at prevalences of 1%, 5%, 10% and 30%. These values span population screening to an enriched specialist-referral setting. For each population, we report the number referred, missed cases, unnecessary confirmatory tests and positive predictive value (PPV). This transport assumes that sensitivity and specificity do not depend on prevalence, which is an approximation outside case–control data.

A post hoc utility sensitivity analysis rescored the saved 50%-budget predictions at FN:FP ratios of 1:1, 2:1, 4:1, 8:1 and 16:1. Models, thresholds and acquisition histories were unchanged. We also propagated each split’s sensitivity and specificity to the hypothetical populations and report the 2.5th and 97.5th percentiles as descriptive split-variability ranges, not population confidence intervals. The analysis code is experiments/revision/reviewer1_operating_sensitivity.py; the derived utility, burden and incremental-burden files are supplied with the revision.

### 4.10. Prevalence-Swap Experiment

To test whether the different gains in the two modalities reflect class balance rather than signal type, the prevalences were exchanged by subsampling. Speech was reduced to the gait prevalence (0.56) by keeping all 64 controls and drawing 83 PD participants. Gait was raised to the speech prevalence (0.75) by keeping all 93 PD participants and drawing 32 controls. A new subsample was drawn for each of the 60 splits, and the component arms were rerun unchanged.

### 4.11. External and Cross-Cohort Validation

For gait, a leave-one-study-out design was used. Each PhysioNet study (Ga, Ju or Si) was held out in turn as an external test cohort, and the other two formed the development pool. Standardization and the RBF bandwidth were estimated from the development pool only. With a fixed test cohort, the 20 repetitions vary only the label-blind initial sample, so intervals describe acquisition variability rather than sampling of test participants. For speech, the complete framework was replicated on the UCI Parkinsons cohort with 60 participant-grouped 70/30 splits. Cross-modal fusion was not attempted, because the gait and speech cohorts contain different people and no participant contributes both signals.

## 5. Conclusions

We developed ClinRisk-LLM to study adaptive cost calibration when labels for PD gait and speech data are limited. A frozen policy, generated without access to test data, chooses the positive-class SVM weight and the priority of negative-side queries from labeled-pool signals. By testing cost-sensitive training and risk-aware acquisition separately, the design avoids attributing improvements from classifier weighting to active learning.

On the two public datasets, joint cAL lowered the common 4:1 cost at half labeling relative to Standard AL. The reduction was 34.1% for gait and 5.7% for speech. Component-wise analyses indicated that classifier weighting accounted for most of the observed reduction, and a symmetric model with a threshold tuned on the labeled set came within 1.3 points of the joint-cAL gait cost. Risk-aware querying added no consistent benefit across splits.

ClinRisk-LLM operationalized the safety specification as a frozen, auditable policy that adjusted risk settings to each modality, maintaining relatively high weights for gait and near-symmetric training for speech. Its incremental effect on cost depended on the dataset and comparison setting. Its gait cost (47.60) was higher than that of fixed cAL (46.10) and slightly lower than that of the deterministic Adaptive Rule (48.03), a difference that was not consistent across splits and reversed under an alternative batch-diversity procedure. Its speech cost (24.69) was slightly higher than that of Standard AL (24.27) and fixed cAL (23.53). Forty policies generated across three LLMs from the reconstructed specification did not match its gait result, and the ranking of the adaptive policies changed with the batch-diversity procedure. These findings support the feasibility of using an LLM to operationalize a safety specification as an adaptive learning policy. The observed cost reductions were driven primarily by cost-sensitive training; the present experiments did not establish a consistent additional reduction in cost from LLM-guided adaptation. Policy replay after freezing and variability during generation should therefore be assessed separately.

Two further results guide the design of future clinical evaluations. First, under the 4:1 loss, the cost-sensitive gait models were roughly level with the trivial policy of referring everyone, and the gait–speech contrast was explained largely by class balance. Second, the lower specificity increases confirmatory-testing demand, whose acceptability depends on target prevalence, test-specific utilities and capacity. The transported low-prevalence scenarios favor alternative operating points under the study’s 4:1 utility.

The next step is to test the framework in external, prevalence-representative cohorts. Utilities elicited from clinical practice should replace the experimental cost ratio. Acquisition policies should also reduce false negatives while maintaining acceptable specificity. Whether digital biosignals could eventually help direct costly molecular confirmation remains a prospective question. Answering it would require multimodal cohorts that pair these recordings with α-synuclein, imaging and genetic labels.

## Figures and Tables

**Table 1:** Mean performance across 60 splits at 50% labeling under the common 4:1 FN:FP loss, with metric-wise best values in bold and the refer-all reference in italics.

Data	Method	FNR	Specificity	Precision	Balanced Accuracy	$C_{4}$
Gait	Random symmetric	0.274	**0.642**	0.734	0.684	77.07
Gait	Standard symmetric	0.237	0.639	**0.739**	**0.701**	69.10
Gait	Query-only	0.251	0.629	0.729	0.689	72.47
Gait	Symmetric + tuned threshold	0.067	0.275	0.627	0.604	46.83
Gait	Cost-only	0.053	0.217	0.609	0.582	46.33
Gait	Joint cAL	**0.049**	0.216	0.609	0.583	**45.57**
*Gait*	*Refer all* ^a^	*0.000*	*0.000*	*0.560*	*0.500*	*44.00*
Speech	Random symmetric	0.021	0.169	0.778	0.574	27.27
Speech	Standard symmetric	0.024	**0.317**	**0.809**	**0.646**	24.42
Speech	Query-only	0.025	**0.317**	0.808	**0.646**	24.69
Speech	Symmetric + tuned threshold	0.016	0.233	0.792	0.609	24.13
Speech	Cost-only	**0.013**	0.239	0.793	0.613	23.09
Speech	Joint cAL	**0.013**	0.246	0.794	0.616	**23.02**
*Speech*	*Refer all* ^a^	*0.000*	*0.000*	*0.747*	*0.500*	*25.33*

^a^ Trivial policy that labels every participant PD; shown as a reference and excluded from bolding.

**Table 2:** Paired split-level differences in $C_{4}$ (A − B) at 50% labeling across the 60 splits. Negative values favor A.

	Gait		Speech	
Comparison (A vs. B)	Median [IQR]	A Lower/Tie	Median [IQR]	A Lower/Tie
Cost-only vs. Standard AL	−20.0 [−34.5, −11.0]	87%/3%	−2.7 [−4.0, 1.3]	55%/7%
Query-only vs. Standard AL	2.0 [−10.0, 15.0]	45%/2%	0.0 [0.0, 0.0]	8%/77%
Joint cAL vs. cost-only	0.0 [−4.5, 4.0]	40%/13%	0.0 [0.0, 0.0]	10%/87%
Joint cAL vs. Standard AL	−21.0 [−34.5, −10.0]	90%/0%	−2.7 [−4.0, 1.3]	55%/7%
Joint cAL vs. refer all	0.0 [−4.0, 8.0]	43%/8%	−2.7 [−6.7, 1.3]	65%/0%
Tuned threshold vs. joint cAL	0.0 [−6.0, 8.0]	40%/12%	1.3 [−1.7, 4.0]	28%/20%
Standard vs. random AL	−6.0 [−20.5, 4.0]	65%/0%	−2.7 [−5.3, 0.0]	67%/12%
ClinRisk-LLM vs. Adaptive Rule	−2.0 [−6.0, 4.5]	52%/8%	0.0 [−1.3, 1.3]	27%/47%
ClinRisk-LLM vs. fixed cAL	0.0 [−4.0, 6.0]	43%/10%	0.0 [−1.3, 4.0]	42%/15%

The adaptive-policy rows use the rank-spreading loop of Table 4. Because the test sets contain 50 (gait) and 75 (speech) participants, $C_{4}$ moves in steps of 2.0 and about 1.3 points, so ties are common.

**Table 3:** Full-pool performance as mean ± one split-level standard deviation, with metric-wise best means in bold.

Data	Method	FNR	Specificity	Balanced Accuracy	$C_{4}$
Gait	Symmetric	0.263±0.079	0.694±0.107	0.716±0.050	72.27±16.01
Gait	Cost-only/joint	0.037±0.043	0.169±0.112	0.566±0.049	44.97±8.37
Gait	Cost-matched threshold	0.048±0.056	0.273±0.128	0.613±0.054	42.67±10.59
Speech	Symmetric	0.022±0.017	0.295±0.096	0.637±0.049	24.36±5.70
Speech	Cost-only/joint	0.012±0.013	0.214±0.088	0.601±0.043	23.38±4.21
Speech	Cost-matched threshold	0.019±0.019	0.278±0.128	0.630±0.061	23.89±5.50

**Table 4:** Mean adaptive-policy performance across 60 splits at 50% labeling, with metric-wise best values in bold.

Data	Policy	$C_{4}$	FNR	Specificity	Balanced Accuracy	Mean $r_{t}$
Gait	Standard AL	72.77	0.258	**0.661**	**0.702**	1.00
Gait	Fixed cAL	**46.10**	**0.048**	0.198	0.575	4.00
Gait	Adaptive Rule	48.03	0.071	0.272	0.600	3.52
Gait	ClinRisk-LLM	47.60	0.067	0.258	0.595	3.47
Speech	Standard AL	24.27	0.022	0.305	0.641	1.00
Speech	Fixed cAL	**23.53**	**0.014**	0.236	0.611	4.00
Speech	Adaptive Rule	24.42	0.023	0.306	0.642	1.13
Speech	ClinRisk-LLM	24.69	0.024	**0.310**	**0.643**	1.03

**Table 5:** Consequences per 1000 people of the mean gait operating points at hypothetical prevalences.

Operating Point	Prevalence	Referred	Detected PD	Missed PD	Unnecessary Tests	PPV	NB(0.2)
Standard AL (50%)	1%	365	7.6	2.4	358	0.021	−0.082
Joint cAL (50%)	1%	786	9.5	0.5	776	0.012	−0.185
Standard AL (50%)	10%	401	76.2	23.8	325	0.190	−0.005
Joint cAL (50%)	10%	801	95.1	4.9	706	0.119	−0.081
Standard AL (50%)	30%	482	228.8	71.2	253	0.475	0.166
Joint cAL (50%)	30%	834	285.2	14.8	549	0.342	0.148
Cost threshold (full pool)	30%	795	285.7	14.3	509	0.359	0.158
Refer all	30%	1000	300.0	0.0	700	0.300	0.125

Sensitivity and specificity are split-level means (Standard AL 0.762/0.639; joint cAL 0.951/0.216; full-pool cost threshold 0.952/0.273). NB(0.2), net benefit at the threshold probability implied by the 4:1 loss.

**Table 6:** External gait cohorts (leave-one-study-out, 20 repetitions) and the independent UCI Parkinsons speech cohort (60 speaker-grouped splits): mean $C_{4}$ at 50% labeling unless stated. UCI performance metrics are calculated per recording, not per speaker.

Method	Gait: Ga Held Out	Gait: Ju Held Out	Gait: Si Held Out	UCI Speech
Test *n* (PD; UCI speakers)	47 (29)	54 (29)	64 (35)	9 (7) per split
Refer all	38.3	46.3	45.3	21.8
Standard AL	54.7	66.2	127.7	28.9
Cost-only	39.5	46.8	62.1	21.7
Joint cAL	39.7	44.9	58.4	22.2
Full-pool cost threshold	35.6	46.0	47.8	28.8
Fixed cAL (adaptive loop)	40.0	46.3	53.9	22.4
Adaptive Rule	41.2	47.7	63.8	24.1
ClinRisk-LLM	39.5	49.7	63.5	25.7
Joint cAL FNR/specificity	0.038/0.208	0.038/0.206	0.080/0.098	0.026/0.350
Symmetric full-pool ROC-AUC	0.77	0.82	0.68	0.68

**Table 7:** Adaptive-policy $C_{4}$ at 50% labeling under rank spreading (as in Table 4) and under kernel *k*-means batch diversity (60 splits).

	Gait		Speech	
Policy	Rank Spreading	k-Means	Rank Spreading	k-Means
Standard AL	72.77	67.10	24.27	24.24
Fixed cAL	46.10	44.47	23.53	23.00
Adaptive Rule	48.03	46.07	24.42	24.44
ClinRisk-LLM	47.60	48.60	24.69	24.42

**Table 8:** Original versus regenerated ClinRisk-LLM policies at 50% labeling (60 splits each).

Data	Policy	$C_{4}$	FNR	Specificity	Mean $r_{t}$
Gait	Original frozen ClinRisk-LLM	47.60	0.067	0.258	3.47
Gait	Sonnet 5 regenerated (*n* = 20), mean (SD)	67.27 (5.10)	0.215 (0.036)	0.565 (0.068)	1.66 (0.39)
Gait	Sonnet 5 regenerated, range	55.90–75.83	0.130–0.274	0.393–0.673	1.18–2.58
Gait	Opus 5.5 (n=10), mean (SD)	55.33 (1.58)	0.128 (0.011)	0.395 (0.033)	2.60 (0.26)
Gait	Haiku 5.5 (n=10), mean (SD)	65.99 (2.39)	0.211 (0.017)	0.573 (0.032)	1.61 (0.23)
Gait	Standard AL/Adaptive Rule	72.77/48.03	0.258/0.071	0.661/0.272	1.00/3.52
Speech	Original frozen ClinRisk-LLM	24.69	0.024	0.310	1.03
Speech	Sonnet 5 regenerated (n=20), mean (SD)	24.78 (0.08)	0.024 (0.000)	0.308 (0.003)	1.00 (0.00)
Speech	Opus 5.5 (n=10), mean (SD)	24.78 (0.16)	0.024 (0.001)	0.309 (0.002)	1.00 (0.00)
Speech	Haiku 5.5 (n=10), mean (SD)	24.77 (0.11)	0.024 (0.000)	0.308 (0.001)	1.00 (0.00)

**Table 9:** Dataset characteristics and analysis units after quality control.

Dataset	*n*	PD	Control	Features
PhysioNet gait	165	93	72	12
Sakar speech	252	188	64	753
UCI Parkinsons speech (recordings)	195	147	48	22

Counts are participants for gait and Sakar speech and recordings for UCI. The UCI dataset contains 32 speaker identifiers (24 PD), whereas its repository description reports 31 participants (23 PD). UCI results are evaluated per recording; splits are grouped by speaker identifier.

**Table 10:** Active-learning and evaluation configuration.

Parameter	Value
Outer split	Stratified 70% pool/30% test
Repeated splits	60 (descriptive, overlapping)
Initial labeled sample	10, selected without label inspection
Query batch/candidate pool	5/25
Reported budgets	10%, 20%, …, 100% of pool
Classifier	RBF-SVM, C=1, pool-derived scale bandwidth
Training weights	Symmetric 1:1 or PD-weighted 4:1
Common evaluation cost	FN:FP = 4:1

## Data Availability

The gait data are available from PhysioNet [21], and the speech data are available from the UCI Machine Learning Repository [23]. The archived analysis code, frozen ClinRisk-LLM policy specification, 40 regenerated policies and aggregate revision results are available at https://github.com/aligunesgit/clinrisk-llm-parkinsons (tag ijms-revision-1, commit ece1b8d; accessed on 21 October 2026). The repository generation archive contains the recovered original user prompt and response, regenerated policies and their results. Additional operating-point sensitivity files and expanded reproduction documentation accompany the present revision; they are subsequent to that archived tag. Policy generation used Claude Code CLI v2.1.241 with its default system configuration; no custom system-prompt override was supplied. The separate reconstruction is the user prompt used for the 40 revision generations.

## Abbreviations

The following abbreviations are used in this manuscript:

AL	Active learning
cAL	Cost-sensitive active learning
$C_{4}$	Weighted cost index at a 4:1 FN:FP ratio
FN	False negative
FNR	False-negative rate
FP	False positive
GRF	Ground-reaction force
LLM	Large language model
OOF	Out-of-fold
PD	Parkinson’s disease
RBF	Radial basis function
SAA	Seed amplification assay
ROC-AUC	Area under the receiver operating characteristic curve
SVM	Support vector machine
